# Novel topoisomerase II/EGFR dual inhibitors: design, synthesis and docking studies of naphtho[2′,3′:4,5]thiazolo[3,2-*a*]pyrimidine hybrids as potential anticancer agents with apoptosis inducing activity

**DOI:** 10.1080/14756366.2023.2205043

**Published:** 2023-05-11

**Authors:** Mai A. E. Mourad, Ayman Abo Elmaaty, Islam Zaki, Ahmed A. E. Mourad, Amal Hofni, Ahmed E. Khodir, Esam M. Aboubakr, Ahmed Elkamhawy, Eun Joo Roh, Ahmed A. Al-Karmalawy

**Affiliations:** a Medicinal Chemistry Department, Faculty of Pharmacy, Port-Said University, Port-Said, Egypt; b Pharmaceutical Organic Chemistry Department, Faculty of Pharmacy, Port-Said University, Port-Said, Egypt; c Pharmacology and Toxicology Department, Faculty of Pharmacy, Port-Said University, Port-Said, Egypt; d Department of Pharmacology and Toxicology, Faculty of Pharmacy, South Valley University, Qena, Egypt; e Department of Pharmacology, Faculty of Pharmacy, Horus University, New Damietta, Egypt; f BK21 FOUR Team and Integrated Research Institute for Drug Development, College of Pharmacy, Dongguk University—Seoul, Goyang, Republic of Korea; g Department of Pharmaceutical Organic Chemistry, Faculty of Pharmacy, Mansoura University, Mansoura, Egypt; h Chemical and Biological Integrative Research Center, Korea Institute of Science and Technology (KIST), Seoul, Korea; i Division of Bio-Medical Science & Technology, University of Science and Technology, Daejeon, Korea; j Pharmaceutical Chemistry Department, Faculty of Pharmacy, Ahram Canadian University, 6th of October City, Giza, Egypt

**Keywords:** Thiazolopyrimidine, naphthoquinone, topoisomerase IIα, EGFR, apoptosis

## Abstract

Topoisomerases II are ubiquitous enzymes with significant genotoxic effects in many critical DNA processes. Additionally, epidermal growth factor receptor (EGFR) plays pivotal role in tumour growth and angiogenesis. A novel series of naphtho[2',3':4,5]thiazolo[3,2-***a***]pyrimidine hybrids have been designed, synthesised and evaluated for their topo IIα/EGFR inhibitory and apoptotic inducer activities. Cytotoxicity of the synthesised hybrids was evaluated against MCF-7, A549 and HCT-116 cell lines. Of the synthesised hybrids, **6i**, **6a** and **6c** experienced superior cytotoxic activity compared to doxorubicin and erlotinib against the tested cancer cells. The molecular mechanism of these hybrids revealed their ability to successfully inhibit topo IIα and EGFR activities in micromolar concentration and may serve as topo II catalytic inhibitor. Moreover, these hybrids significantly arrested cell cycle at G2/M phase together with increased p53, caspae-7, caspase-9 levels and Bax/Bcl-2 ratio. The synthesised hybrids showed efficient binding pattern in molecular docking study and have acceptable drug likeness characters.

## GRAPHICAL ABSTRACT

**Figure:**
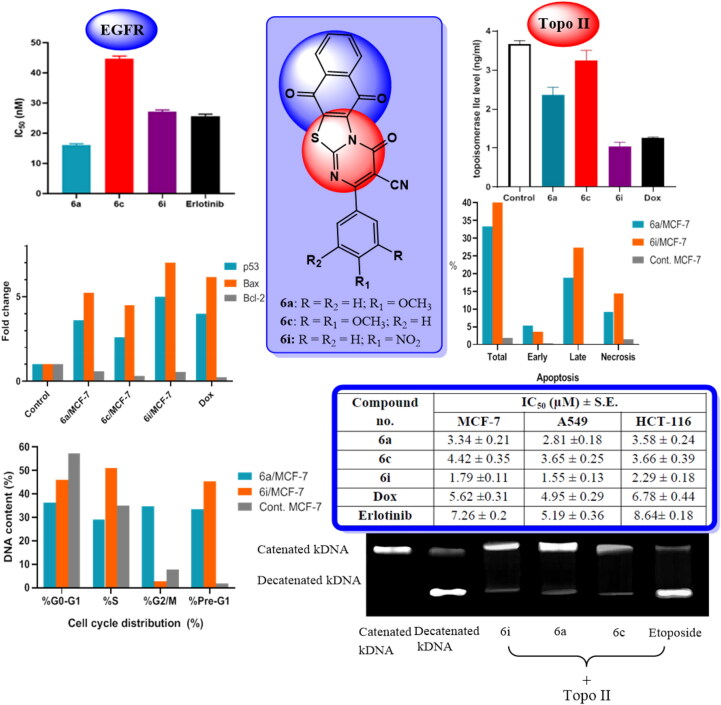

## Introduction

Cancer is one of the most dreaded diseases and the second leading cause of death worldwide after cardiovascular diseases^1^. Despite the rapid progress in discovering novel chemotherapeutic strategies for the treatment of different types of cancer, cancer prevalence represents a rapidly aggravating problem all over the world^2^. Moreover, the lack of selectivity, efficacy, safety and multi-drug resistance in currently available chemotherapeutic drugs represent hurdles against prosperity of cancer chemotherapy^3^. Consequently, there is an urgent demand to explore novel chemotherapeutic agents with high selectivity, efficacy and minimum side effects. One of the most important targets of antineoplastic drugs is DNA topoisomerases^4–6^.

DNA topoisomerases are essential nuclear enzymes that maintain the topological changes of DNA and play key role in catalysing DNA replication, proliferation, transcription, recombination, repair, chromosome condensation and segregation^4^^,^^7^^,^^8^. Additionally, most of available anticancer regimens based on using at least one member of topoisomerase inhibitors^6^. However, DNA topoisomerases are generally classified into topoisomerase I (topo I) and topoisomerase II (topo II)^9^. Topo I possesses its functions by cleaving only one strand of DNA, while type II cleaves both DNA strands^10^. In the same context, topo II catalysis requires two cofactors ATP and Mg^+2^ in order to carry out its double-stranded DNA passage reaction^11^.

Notably, human topo II encodes two isoforms (topo Iiα and topo Iiβ); both share 70% sequence similarity^12^. Topo Iiα isoform is expressed in highly proliferating and tumour cells and implicated in cell cycle events, in contrast topo Iiβ isoform is predominantly expressed in non-proliferating cells such as neurons and cardiac cells^13^. Therefore, topo Iiα is considered as more important target than topo Iiβ for the development of chemotherapeutic agents and it is already targeted by wide range of anticancer agents^14^.

**Figure 1.**
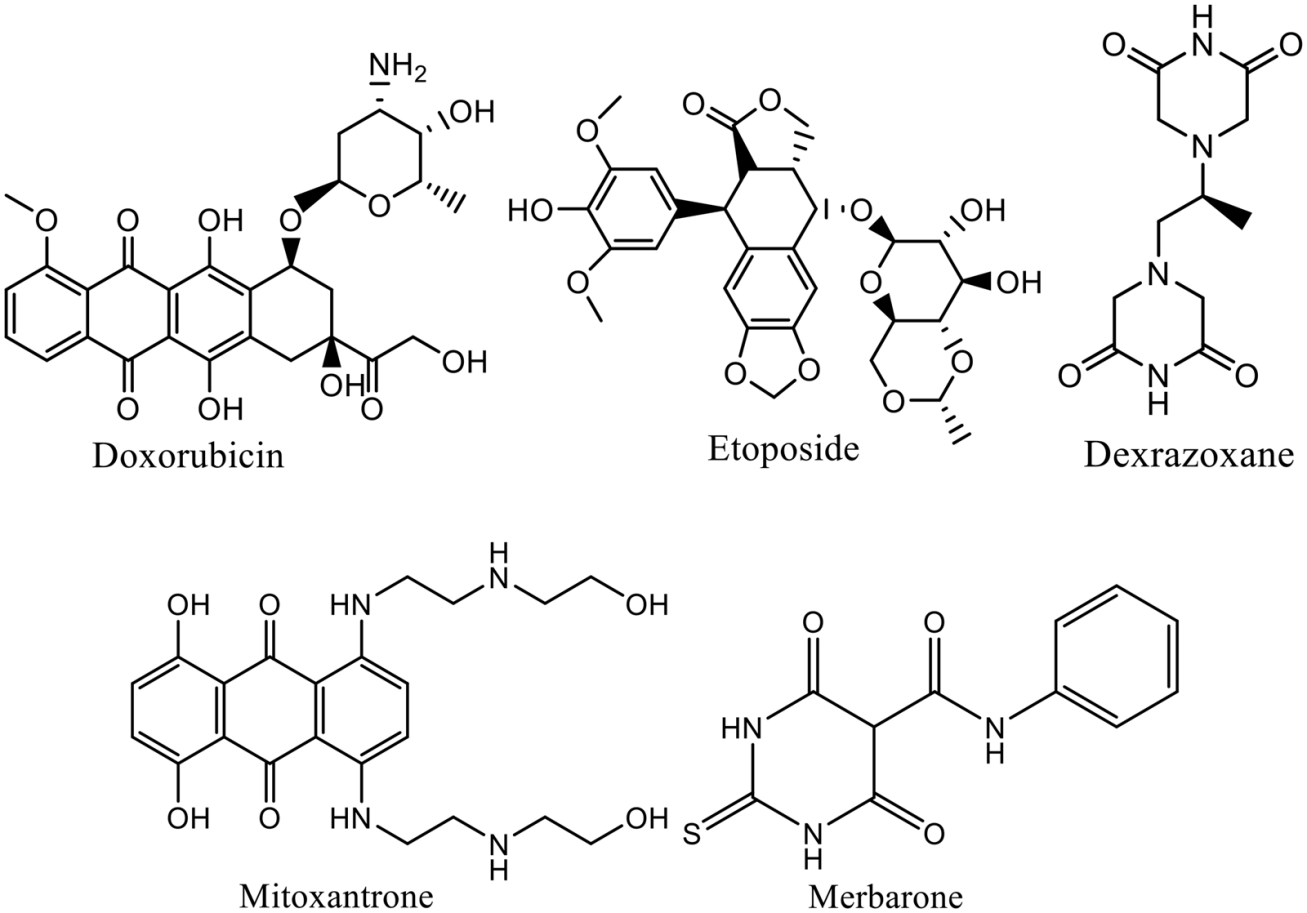
Examples for anticancer agents targeting topoisomerase II.

Topo II inhibitor drugs can be classified according to mechanism of action into two classes^15^. The first class is topo II poison, such as doxorubicin, mitoxantrone and etoposide, which are regarded as the most important topoisomerase inhibitors especially against kidney, breast and lung cancers and they are considered as lead compounds for developing more beneficial anticancer agents^16–18^. This class inhibits the re-ligation step of cleaved DNA molecules resulting in DNA damage and preventing its replication and transcription^19^. The second class is topo II catalytic inhibitors, such as merbarone, novobiocin, and dexrazoxane, which act on inhibiting certain catalytic step of the topo II catalytic cycle, including DNA cleavage and prevention of ATP hydrolysis (Figure 1)^20^. Furthermore, topo II poison inhibitors suffering from tumour resistance and life-threatening toxic effects^21^. Therefore, seeking for newer topo II inhibitors are ongoing with the hope of enhanced potency and overcoming the aforementioned side effects.

**Figure 2.**
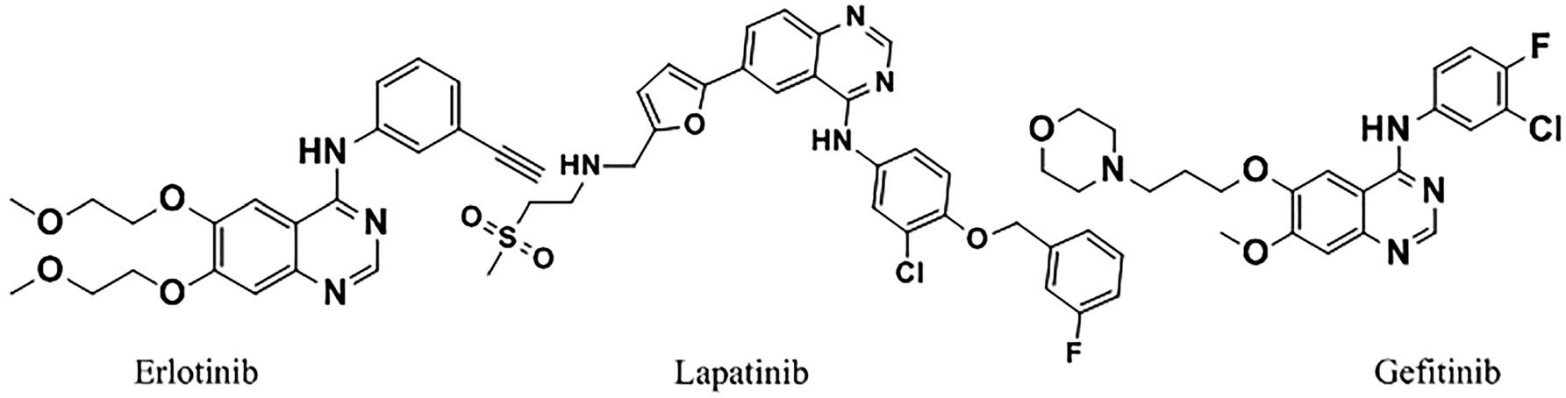
Clinically approved EGFR anticancer drugs.

Of considerable interest, protein tyrosine kinases are considered one of the most important protein families that contribute to many diseases, particularly cancer, as they are involved in almost every aspect of the cellular processes^22^. Among them, epidermal growth factor receptor (EGFR) which plays a key role in cell proliferation, survival, growth, angiogenesis, differentiation and metastasis^23^. It is over-expressed in a significant number of human tumours specifically non-small cell lung cancer (NSCLC) and breast cancer^24^. In view of this, targeting EGFR is considered an efficient goal for the development of new anti-cancer agents that inhibit tumour angiogenesis and consequently tumour growth. Several EGFR inhibitors have been reported, including the Food and Drug Administration (FDA)-approved erlotinib, lapatinib and gefitinib^25^^,^^26^ (Figure 2).

Worthily, thiazolo[3,2-***a***]pyrimidine are interesting group of heterocyclic compounds that have been contributed efficiently in the field of anticancer research as useful chemotherapeutic agents. Additionally, this group endowed with myriad of biological activities such as antimicrobial^27^, antioxidant^28^, antiparkinsonian^29^, antiviral^30^ anti-inflammatory^31^ and anti-HSV-1^32^. Meanwhile, some of the newly synthesised series of thiazolo[3,2-***a***]pyrimidines and thiazolopyrimidine hydrobromides derivatives have shown considerable cytotoxic activity with significant topo II inhibitory activity^33–35^. Furthermore, a series of thiazolo[4,5-d]pyrimidines were described as antitumor agents with remarkable EGFR inhibitory activity^36–38^. On the other hand, naphthoquinone-based moieties are engaged in synthesising scaffolds with potent cytotoxic activity^39–42^. Additionally, novel naphthoquinone-hydrazinotriazolothiadiazine analogues exhibited potential inhibitory profile activity against topo Iiβ^43^.

**Figure 3.**
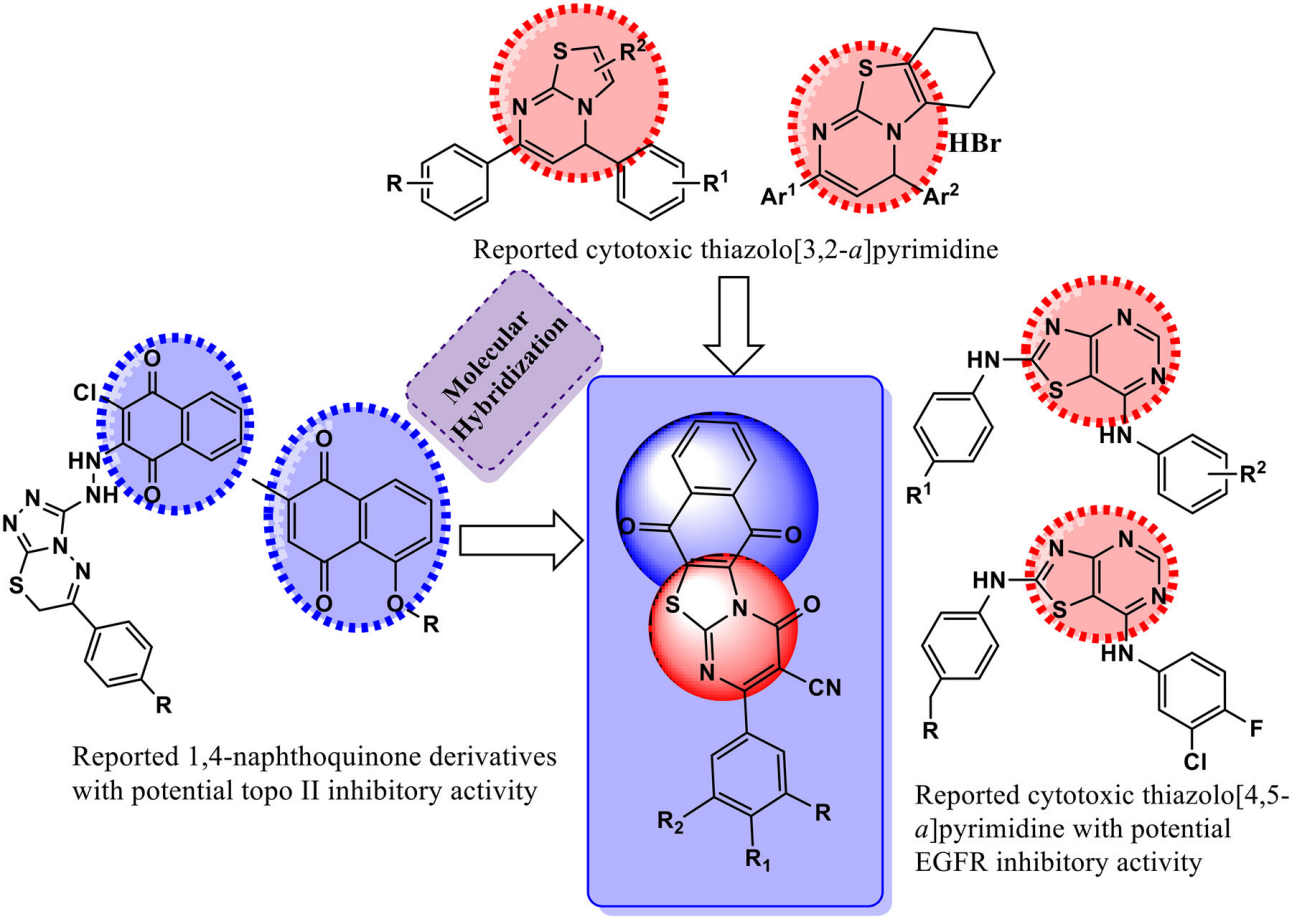
Diagram represents design of novel hybrids based upon thiazolo[3,2-*a*] pyrimidine and 1,4-naphthoquinone moieties as dual topo II/EGFR inhibitor.

Therefore, in an attempt to discover novel topo Iiα/EGFR inhibitors, the present work aims to design novel series of naphtho[2′,3′:4,5]thiazolo-[3,2-***a***]pyrimidine hybrids via molecular hybridisation of the two bioactive pharmacophores thiazolo[3,2-***a***]pyrimidine and naphthoquinone into a single candidate template for the purpose of synergistic anticancer activity (Figure 3). The synthesised hybrids have been tested as potential anticancer therapeutics against MCF-7, A549 and HCT-116 cell lines. Furthermore, hybrids eliciting promising anticancer activity were further investigated as topo Iiα/EGFR inhibitors. Additionally, the cytotoxic activity of the most powerful compounds was clarified by assessing cell cycle, p53, caspases 7 and 9 levels, and Bax/Bcl2 ratios. Meanwhile, the physicochemical properties and the molecular docking study for the synthesised hybrids against the conserved ATPase domain of topo-Iiα and EGFR were carried out to reveal mutual binding modes of the synthesised hybrids into DNA minor groove and specify the structural features essential for DNA recognition of these hybrids.

## Material and methods

### Synthesis of lead compounds

Chemicals and solvents used in the preparation of the target compounds are of commercial grade, and purchased from Alfa Aesar, Cambrian chemicals, Sigma-Aldrich, Acros Organics, Fluka, Merck, and El-Nasr Pharmaceutical Chemicals Companies. All chemicals were used as received.

Melting points were determined on Mel-Temp hot stage apparatus and are uncorrected. Reactions were monitored by thin-layer chromatography on Merck silica gel PF_254_ plates and spots were visualised with UV light at 254 nm. IR spectra were recorded on a Bruker FT-IR spectrophotometer. ^1^H NMR and ^13^C NMR spectra were carried out on a Bruker apparatus operating at 400 MHz and 100 MHz, respectively using TMS as internal reference. Chemical shifts (δ values) are given in parts per million (ppm) from TMS using DMSO-d_6_ (2.50 ppm) as solvent and coupling constants (*J*) in Hz. Splitting patterns are designated as follows: s, singlet; bs, broad singlet; d, doublet; t, triplet; m, multiplet. EI-MS: Mass spectra were carried out on direct probe controller inlet part to single quadruple mass analyser in (Thermo Scientific Gcms) Model (ISQ LT) using Thermo X-Calibur Software at the Regonal Centre for Mycology and Biotechnology (RCMB) Al-Azhar University, Nasr City, Cairo. Elemental analyses were performed on Perkin–Elmer 2400 CHN Elemental analyser by the Microanalytical Centre, Faculty of Science at the University of Cairo and are within ± 0.4% of calculated values unless otherwise specified.

2-Mercapto-6-oxo-4-aryl-1,6-dihydropyrimidine-5-carbonitriles **4a-I** were synthesised according to the reported procedures^44–47^.

#### General procedure for the synthesis of 2-aryl-4,6,11-trioxo-6,11-dihydro-4H-naphtho[2′,3′:4,5]thiazolo-[3,2-a]pyrimidine-3-carbonitriles 6a-i

A mixture of the appropriate 2-mercapto-6-oxo-4-aryl-1,6-dihydropyrimidine-5-carbonitril**es 4a-i** (1 mmol) and 2,3-dichloro-1,4-naphthoquinone **(5)** (1 mmol) was dissolved in 5 ml DMF at rt. (32 °C). The mixture was left at rt. for 12 h. The solid formed was filtered off, washed with hot ethanol and recrystallized from DMF to give the titled compounds.

##### 2–(4-Methoxyphenyl)-4,6,11-trioxo-6,11-dihydro-4*H*-naphtho[2′,3′:4,5]- thiazolo[3,2-*a*]pyrimidine-3-carbonitrile (6a)

Orange solid; yield: 0.38 g, 94%; mp. >300 °C. IR(KBr, υ, cm^−1^), 3001(Ar-CH), 2978, 2941, 2814(Aliph-CH), 2220 (CN), 1701(CO), 1662 (C = N), 1542, 1474 (C = C). ^1^H NMR (400 MHz, DMSO-d_6_) δ= 7.88(d, 1H, *J* = 8.1 Hz, Ar-H), 7.48(d, 1H, *J* = 8.1 Hz, Ar-H), 7.36(t, 1H, *J* = 8.3 Hz, Ar-H), 7.30(t, 1H, *J* = 8.3 Hz, Ar-H), 7.18(d, 2H, *J* = 8.1, Hz, Ar-H), 6.96(d, 2H, *J* = 8.1 Hz, Ar-H), 4.83(s, 3H, OCH_3_).^13^C NMR (100 MHz, DMSO-d_6_) δ = 177.5 (CO), 173.8 (CO), 159.8 (CO), 156.2, 152.4, 141.8, 137.9, 137.5, 135.6, 133.2, 132.0, 131.0, 130.0 (2 C), 127.1, 125.1, 122.6, 117.2 (2 C), 116.0, 99.1, 55.8 (OCH_3_). MS m/z (rel. abundances, %) = 414(M^+1^, 55), 413(M^+^, 100), 386(88), 362(41), 338(66), 294(57), 260, (62), 255(48), 240(40), 228(47), 188(57), 184(35), 170(42), 149(77), 120(49), 104(46), 76(51). Anal. Calcd for C_22_H_11_N_3_O_4_S (413.41), C 63.92, H 2.68, N 10.16, S 7.76; Found: C 63.71, H 2.91, N 10.04.

##### 2–(4-Fluorophenyl)-4,6,11-trioxo-6,11-dihydro-4*H*-naphtho[’',’':4,5]thiazolo- [3,2-*a*]pyrimidine-3-carbonitrile (6b)

Yellow solid; yield: 0.36 g, 86%; mp. >300 °C. IR(KBr, υ, cm^−1^), 3050, 3007 (Ar-CH), 2215 (CN), 1707 (CO), 1675 C = N), 1584, 1452 (C = C). ^1^H NMR (400 MHz, DMSO-d_6_) δ= 8.24 (d, 2H, *J* = 8.1 Hz, Ar-H), 8.11 (d, 2H, *J* = 8.1 Hz, Ar-H), 7.96 (*m*, 2H, Ar-H), 7.34 (d, 2H, *J* = 8.1 Hz, Ar-H). ^13^C NMR (100 MHz, DMSO-d_6_) δ = 180.0 (CO), 179.3 (CO), 163.8 (CO), 162.1, 148.0, 142.1, 140.8, 140.0, 138.3, 137.0, 134.8, 134.2, 133.1, 132.2 (2 C), 131.2, 128.6, 121.6, 118.7(2 C), 95.0. MS *m/z* (rel. abundances, %) = 402(M^+1^, 23), 401(M^+^, 100), 382(25), 376(41), 352(57), 289(75), 260(58), 255(76), 244(19), 216(41), 202(43), 188(39), 104(20), 77(27). Anal. Calcd for C_21_H_8_ FN_3_O_3_S (401.37), C 62.84, H 2.01, F 4.73, N 10.47, S 7.99; Found: C 63.01, H 1.91, N 10.34.

##### 2–(3,4-Dimethoxyphenyl)-4,6,11-trioxo-6,11-dihydro-4*H*-naphtho[’',’':4,5]- thiazolo[3,2-*a*]pyrimidine-3-carbonitrile (6c)

Grey solid; yield: 0.41 g, 92%; mp. 185–187 °C. IR(KBr, υ, cm^−1^), 3171, 3107 (Ar-CH), 2971, 2932 (Aliph-CH), 2221 (CN), 1709 (CO), 1671 (C = N), 1557, 1473 (C = C). ^1^H NMR (400 MHz, DMSO-d_6_) δ = 8.12 (*m*, 2H, Ar-H), 8.01(d,1H, *J* = 8.2 Hz, Ar-H), 7.92(d, 1H, *J =* 8.2 Hz, Ar-H), 7.72(d, 1H, *J* = 8.2 Hz, Ar-H), 7.53(d, 1H, *J* = 8.1 Hz, Ar-H), 7.08(d, 1H, *J* = 8.2 Hz, Ar-H), 7.04 (s, 1H, Ar-H), 3.88(s, 3H, OCH_3_). 3.83(s, 3H, OCH_3_). MS m/z (rel. abundances, %) = 444(M ^+ 1^, 24), 443(M^+^, 100), 431(64), 420(76), 417(68), 381(80), 371(94), 325(35), 286(51), 256(67), 205(41), 187(38), 157(29), 123(54), 104(56), 77(37). Anal. Calcd for C_23_H_13_N_3_O_5_S (443.43); C 62.30, H 2.95, N 9.48, S 7.23; Found:, C 62.52, H 2.80, N 9.54.

##### 2–(4-Bromophenyl)-4,6,11-trioxo-6,11-dihydro-4*H*-naphtho[’',’':4,5]thiazolo- [3,2-*a*]pyrimidine-3-carbonitrile (6d)

Violet solid; yield: 0.41 g, 88%; mp. >300 °C. IR(KBr, υ, cm^−1^), 3139, 3009 (Ar-CH), 2206 (CN), 1704 (CO), 1669 (C = N), 1557, 1468 (C = C). ^1^H NMR (400 MHz, DMSO-d_6_) δ = 7.94(m, 1H, Ar-H), 7.90(d, 1H, *J* = 8.1 Hz, Ar-H), 7.81(*m*, 2H, Ar-H), 7.39(d, 2H, *J* = 8.1 Hz, Ar-H), 7.26(d, 2H, *J* = 8.1 Hz, Ar-H). ^13^C NMR (100 MHz, DMSO-d_6_) δ = 179.0 (CO), 177.5 (CO), 173.3 (CO), 159.2, 155.1, 144.0, 139.8, 137.8, 137.0, 136.2, 131.8, 131.4, 131.0 (2 C), 130.4, 128.1, 126.1 (2 C), 119.6, 114.1, 96.0. MS *m/z* (rel. abundances, %) = 464(M ^+ 2^, 38), 462(M^+^, 51), 434(100), 401(92), 399(92), 356(35), 306(43), 273(45), 234(61), 228(22), 189(29), 177(53), 157(41), 104(31), 77(25). Anal. Calcd for C_21_H_8_BrN_3_O_3_S (462.28): C 54.56, H 1.74, Br 17.28, N 9.09, S 6.94; Found: C 54.77, H 1.92, N 9.24.

##### 2–(4-Chlorophenyl)-4,6,11-trioxo-6,11-dihydro-4*H*-naphtho[’',’':4,5]thiazolo- [3,2-*a*]pyrimidine-3-carbonitrile (6e)

Pale orange solid; yield: 0.38 g, 92%; mp. >300 °C. IR(KBr, υ, cm^−1^), 3044, 3002 (Ar-CH), 2217 (CN), 1705 (CO), 1671 (C=N), 1564, 1554 (C=C). ^1^H NMR (400 MHz, DMSO-d_6_) δ = 8.21(*m*, 2H, Ar-H), 7.92(d, 2H, *J* = 8.1 Hz, Ar-H). 7.95(d, 2H, *J* = 8.1 Hz, Ar-H), 7.35(m, 2H, Ar-H). MS m/z (rel. abundances, %) = 419(M ^+ 1^, 25), 418(M^+^, 60), 382(34), 361(45), 255(100), 228(38), 188(41), 168(30), 159(45), 104(44), 77(14). Anal. Calcd for C_21_H_8_ClN_3_O_3_S (417.82): C 60.37, H 1.93, Cl 8.49, N 10.06, S 7.67; Found: C 60.10, H 2.22, N 9.89.

##### 4,6,11-Trioxo-2–(3,4,5-trimethoxyphenyl)-6,11-dihydro-4*H*-naphtho[’',’':4,5]- thiazolo[3,2-*a*]pyrimidine-3-carbonitrile (6f)

Yellow solid; yield: 0.42 g, 89%; mp. >300 °C. IR(KBr, υ, cm^−1^), 3137, 3009 (Ar-CH), 2973, 2930 (Aliph-CH), 2202 (CN), 1707 (CO), 1680 (C=N), 1539, 1410 (C=C). ^1^H NMR (400 MHz, DMSO-d_6_) δ = 7.97(d, 1H, *J* = 8.2 Hz, Ar-H), 7.87(m, 2H, Ar-H), 7.41(d, 1H, *J* = 8.2 Hz, Ar-H), 6.51(s, 2H, Ar-H), 3.88(s, 3H, OCH_3_), 3.85(s, 6H, 2OCH_3_). ^13^C NMR (100 MHz, DMSO-d_6_) δ = 181.0 (CO), 178.6 (CO), 169.7 (CO), 159.4, 154.1, 150.6 (2 C), 143.0, 136.8, 136.4, 135.2, 134.0, 133.1, 131.1, 129.8, 124.5, 112.1, 110.5 (2 C), 108,3, 97.2, 60.4 (OCH_3_), 54.1 (2 OCH_3_). MS m/z (rel. abundances, %) = 475(M^+2^, 21), 473(M^+^, 100), 445(33), 372(12), 318(29), 316(31), 245(34), 239(31), 217(25), 157(43), 104(37), 77(38). Anal. Calcd for C_24_H_15_N_3_O_6_S (473.46): C 60.88, H 3.19, N 8.88, S 6.77; Found: C 60.59, H 3.32, N 8.74.

##### 4,6,11-Trioxo-2-phenyl-6,11-dihydro-4*H*-naphtho[’',’':4,5]thiazolo[3,2-*a*]- pyrimidine-3-carbonitrile (6 g)

Brown solid; yield: 0.34 g, 90%; mp. >300 °C. IR(KBr, υ, cm^−1^), 3172, 3105, 3074 (Ar-CH), 2208 (CN), 1711 (CO), 1666 (C=N), 1553, 1440 (C=C). ^1^H NMR (400 MHz, DMSO-d_6_) δ = 8.05(m, 2H, Ar-H), 7.94(d, 1H, *J* = 8.1 Hz, Ar-H), 7.87(d, 1H, *J* = 8.1 Hz, Ar-H), 7.13(d, 2H, *J* = 8.1 Hz, Ar-H), 7.04 and 6.94(2t, 3H, *J* = 8.3 Hz, Ar-H). ^13^C NMR (100 MHz, DMSO-d_6_) δ = 179.1 (CO), 177.0 (CO), 171.2 (CO), 158.6, 156.6, 144.7, 138.4, 137.1, 134.6, 133.8, 133.0, 132.4, 130.2, 124.1 (2 C), 124.0, 122.1 (2 C), 115.5, 112.9, 98.2. MS *m/z* (rel. abundances, %) = 384(M ^+ 1^, 18), 383(M^+^, 100), 355(21), 306(55), 256(54), 228(57), 177(23), 157(44), 140(22), 104(40), 77(47). Anal. Calcd for C_21_H_9_N_3_O_3_S (383.38): C 65.79, H 2.37, N 10.96, S 8.36; Found: C 65.52, H 2.33, N 11.23.

##### 2–(4-(Dimethylamino)phenyl)-4,6,11-trioxo-6,11-dihydro-4*H*-naphtho- [’',’':4,5]thiazolo[3,2-*a*]pyrimidine-3-carbonitrile (6h)

Green solid; yield: 0.39 g, 92%; mp. >300 °C. IR(KBr, υ, cm^−1^), 3054, 3015 (Ar-CH), 2909, 2865, 2800 (Aliph-CH), 2210 (CN), 1705 (CO), 1677 (C=N), 1519, 1429 (C=C). ^1^H NMR (400 MHz, DMSO-d_6_) δ = 7.95(*m*, 2H, Ar-H), 7.81(2d, 2H, *J* = 8.2 Hz, Ar-H), 7.17(d, 2H, *J* = 8.2 Hz, Ar-H), 6.98(d, 2H, *J* = 8.2 Hz, Ar-H), 3.11(s, 6H, 2CH_3_). MS m/z (rel. abundances, %) = 428(M ^+ 2^, 52), 426(M^+^, 100), 401(32), 384(11), 362(42), 271(39), 256(77), 228(48), 171(41), 158(51), 132(23), 120(49), 104(50), 77(56), 44(23), Anal. Calcd for C_23_H_14_N_4_O_3_S (426.45): C 64.78, H 3.31, N 13.14, S 7.52; Found: C 64.49, H 3.42, N 13.24.

##### 2–(4-Nitrophenyl)-4,6,11-trioxo-6,11-dihydro-4*H*-naphtho[’',’':4,5]thiazolo- [3,2-*a*]pyrimidine-3-carbonitrile (6i)

Brown solid; yield: 0.36 g, 85%; mp. 162–164 °C. IR(KBr, υ, cm^−1^), 3074, 3011 (Ar-CH), 2971, 2923, 2866 (Aliph-CH), 2221 (CN), 1709 (CO), 1666 (C=N), 1521, 1479 (C=C). ^1^H NMR (400 MHz, DMSO-d_6_) δ = 8.35(d, 2H, *J =* 8.1 Hz, Ar-H), 8.03(*m*, 2H, Ar-H), 7.50(d, 2H, *J* = 8.1 Hz, Ar-H), 7.02(d, 2H, *J* = 8.1 Hz, Ar-H). ^13^C NMR (100 MHz, DMSO-d_6_) δ = 182.1 (CO), 178.2 (CO), 167.2 (CO), 165.1, 152.2, 143.1, 141.2, 139.0, 137.9, 137.2, 136.6, 136.1 (2 C), 132.1, 125.1, 123.4 (2 C), 121.9, 121.3, 111.2, 99.1. MS m/z (rel. abundances, %) = 429(M ^+ 1^, 14), 428(M^+^, 69), 399(28), 382(27), 312(51), 304(39), 273(100), 256(47), 228(31), 172(49), 136(31), 122(33), 104(41), 77(27). Anal. Calcd for C_21_H_8_N_4_O_5_S (428.38): C 58.88, H 1.88, N 13.08 S 7.49; Found: C 59.08, H 1.91, N 12.87.

### Assessment of anti-cancer activity

#### Cell culture

Breast carcinoma (MCF-7), lung carcinoma (A549) and colorectal carcinoma (HCT-116) were purchased from American Type Cell Culture Collection (ATCC, Manassas, USA). MCF-7 and A549 cell lines were cultured in Dulbecco’s Modified Eagle’s Medium (DMEM, Invitrogen/Life Technologies), while HCT-116 cell line was maintained in RPMI1640 (HyClone). The culture media were supplemented with 10% heat inactivated foetal bovine serum (FBS, HyClone, Thermo Scientific) and antibiotics (100 U/ml penicillin and 100 µg/ml streptomycin, HyClone, Thermo Scientific). The cells were incubated in a humidified atmosphere containing 5% CO_2_ (Heracell VIOS CO_2_ incubator, Thermo Scientific) in air at 37 °C.

#### In vitro anti-proliferative activity

The *in vitro* anti-proliferative activity of the synthesised hybrids (**6a-i**) was evaluated using MTT (3–(4, 5-dimethylthiazol-2-yl)-2, 5-diphenyltetrazolium bromide) assay, against three cancer cell lines, MCF-7, A549 and HCT-116. Cancerous cells were incubated with different concentrations of the synthesised hybrids and reference compounds (0.4 µM, 1.6 µM, 6.3 µM, 25 µM and 100 µM) for 24 h. MTT reagent, at volume equal to 10% of the culture medium volume, was added to the cultured cells and to the blank wells. The culture plates were re-incubated for 2–4 h depending on cell type and density. After the incubation period, culture plates were restored from the incubator and the obtained formazan crystals were dissolved in acidified isopropanol with continuous shaking using plate shaker (MaxQ 2000, Thermo Fisher Scientific Inc., MI, USA) at room temperature. Absorbance was measured spectrophotometrically at wavelength 570 nm using a plate reader (Stat FaxR 4200, Awareness Technology, Inc., FL, USA) [48]. Cell viability was calculated as percentage of control and the concentration that inhibits 50% of maximum cell proliferation was determined and expressed as IC_50_ using Graph Pad Prism 5 software (Graph Pad software Inc., CA, USA). Three independent experiments were performed for each compound and Mean ± SE were calculated.

#### Topoisomerase iIα inhibitory activity

Topoisomerase iIα inhibitory activity of **6a, 6c** and **6i** hybrids was assessed using the human DNA topoisomerase Elisa kit (Abcam, Japan, Recombinant Human Topoisomerase II alpha protein). **6a, 6c** and **6i** hybrids or doxorubicin were added to the wells and incubated for 1 h at 37 °C. Biotin-conjugated antibody was added to each well followed by incubation at 37 °C for 1 h, then the plates were aspirated. Avidin conjugated Horseradish Peroxidase (HRP avidin) solution was added to each well, incubated again at 37 °C for 1 h. TMB substrate was added to the wells and the plate was incubated at 37 °C for 30 min at dark place. The absorbance was read using spectrophotometer at a wavelength of 450 nm.

#### DNA topo II inhibition assay

Using the procedure outlined in topoisomerase II Drug Screening Kit (Topogen, USA), the effect of **6a**, **6c** and **6i** on topoisomerase II inhibition and the effectiveness of catenated/decatenated kDNA formation were examined. The reaction mixture includes 10 mM MgCl_2_, 50 mM Tris-HCl, 0.5 mM dithiothreitol, 30 mg/ml bovine serum albumin, 200–300 ng of kDNA and topoisomerase II. Test compounds were added to reaction mixture and etoposide used as reference standard. After the reaction samples were incubated for 30 min at 37 °C, the reaction was stopped by adding 2 ml of a stop buffer containing 10% (w/v) SDS and 2 ml of 0.5 mg/ml proteinase followed by incubating the mixture for 10 min at 37 °C. The reaction mixture was separated by 1% agarose gel and examined after being stained with ethidium bromide.

#### EGFR inhibitory assay

The kinase activity of EGFR was assessed for **6a, 6c** and **6i** hybrids using EGFR kinase assay kit (BPS Bioscience, San Diego, CA, USA) based on the manufacturer’s instructions. Three independent experiments were performed for each compound and Mean ± SE were calculated.

#### Determination of Bax and Bcl-2 levels

MCF-7 cells were cultured in DMEM supplied with 10% FBS and incubated in 5% CO_2_ in air at 37 °C. After incubation with the synthesised hybrids (**6a, 6c** and **6i**), at their IC_50_ concentrations, for 24 h, MCF-7 were lysed with extraction buffer. Cell lysate was diluted using standard diluent buffer over the range of assay. Bax and Bcl-2 content in the lysate was assessed using human Bax ELISA kit (DRG, USA) and Bcl-2 ELISA Kit (Zymed Laboratories, Invitrogen Immuno-detection, Canada), respectively, according to the manufacturer’s instructions for each kit. The results were expressed as Mean ± SE of three independent experiments.

#### Determination of p53 level

p53 level was determined in MCF-7 cell line using Human p53 ELISA Kit (Sigma Aldrich, St. Louis, MO, USA). The cells were lysed using cell extraction buffer. Cell lysate was diluted with standard diluent buffer, then added to p53 monoclonal antibody pre-coated 96-wells plate. The plate incubated for 2 h at room temperature. Anti-p53 detection antibody was added to the plate and re-incubated at room temperature for 1 h. The plate washed four times using wash buffer followed by addition of anti-rabbit IgG-Horseradish Peroxidase (HRP) working solution. The plate incubated at room temperature for 30 min. Chromogen TMB (tetramethylbenzidine) was added to induce colour reaction. The absorbance of the plate was read at 450 nm using microplate reader (Bio-Rad Laboratories, Hercules, CA, USA). Three independent experiments were performed for each compound and Mean ± SE were calculated.

#### Caspase 7 and 9 assay

The effect of **6a, 6c** and **6i** hybrids on caspase-7 and −9 levels in MCF-7 cell line was measured using Human Caspase-7 ELISA Kit (BioVision Inc., Milpitas, CA 95035 USA) and Human Caspase-9 ELISA Kit (EIA-4860, DRG, USA), respectively, and according to the manufacturer’s instructions. The results were expressed as Mean ± SE of three independent experiments.

#### In vitro DNA flow Cytometry assay

MCF-7 cells were seeded at density of 2 × 10^5^ into each well of a 6- well plate. The cells were maintained in Dulbecco’s Modified Eagle’s Medium (DMEM), supplied with 10% foetal bovine serum, and incubated in a humidified atmosphere containing 5% CO_2_ in air for 24 h at 37 °C. The incubation medium was replaced with a fresh one containing the synthesised hybrids **6a** and **6i** at their IC_50_ in DMSO (1% v/v). The cell plates were incubated for 24 h. The cells were washed twice with cold phosphate buffered saline (PBS) then fixed with 70% ice-cold ethanol. Cells washed with PBS at 37 °C for 30 min and recovered by centrifugation at 2000 rpm for 5 min. Cells were stained using DNA fluorochrome propidium iodide (PI). The plates were incubated at room temperature for 20 min in a dark place. Then the cells were investigated with a FACS Calibre flow cytometer (Becton Dickinson, Heidelberg, Germany)^48^.

#### Annexin V-FITC/PI staining assay

MCF-7 cells were incubated at a density of 4 × 10^6^/well with synthesised compounds **6a** and **6i** at their IC_50_ for 24 h. Cells underwent three washing cycles using ice cold PBS, then suspended in PBS. Cell apoptosis was detected by Annexin V-FITC Apoptosis Detection Kit (BioVisionResearch Products, USA). The cells were stained using PI staining solution, Annexin V-FITC and incubated for 15 min at room temperature in a dark place. Cells were investigated by flow cytometry (Ex = 488 nm; Em = 530 nm) using FACS calibre (Becton Dickinson, Heidelberg, Germany)^48^.

#### Physicochemical and pharmacokinetic properties prediction

Physicochemical and pharmacokinetic properties as well as medicinal chemistry friendliness and drug-like nature of the synthesised compounds were calculated and predicted based on online pkCSM software.

#### Statistical analysis

Results were expressed as means ± standard error of the mean (SEM). GraphPad Prism® was used for statistical calculations (GraphPad Software, San Diego California USA).

#### Molecular docking

Molecular docking study of the synthesised hybrids (**6a-i**), Dox and erlotinib was performed using MOE software. Ligands were built into the builder interface of the MOE program and their energies were minimised until a RMSD (root mean square deviations) gradient of 0.01 kcal/mol and RMS (Root Mean Square) distance of 0.1 Å with MMFF94× (Merck molecular force field 94×) force-field and the partial charges were automatically calculated. The X-ray crystallographic structure of ATP- active sites of Dox and EGFR (PDB code: 1ZXM and1XKK, respectively), were downloaded from protein data bank (www.rcsb.org). The enzymes were prepared, the hydrogens were added then the atoms connection and type were checked with automatic correction. The obtained poses were studied and the poses showed best ligand-enzyme interactions were selected and stored for energy calculations.

## Result and discussion

### Chemistry

**Scheme 1.**
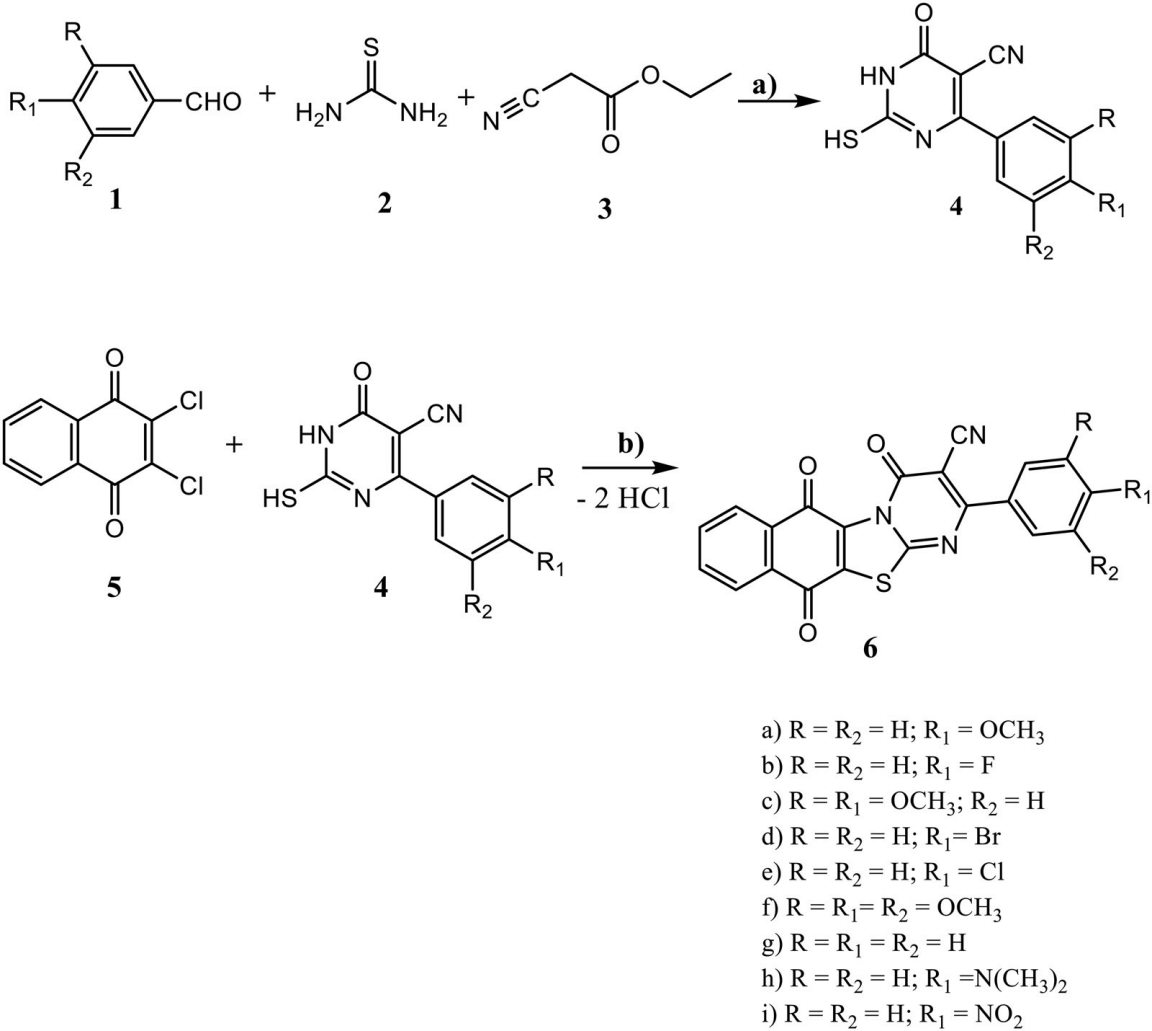
Synthesis of the target hybrids **6a-i**. Reagents and conditions: a) K2CO3, C2H5OH, reflux, 2h. b) DMF , rt., 12 h.

6-Aryl-2-mercapto-1,6-dihydropyrimidine-5-carbonitriles **4a-i** were synthesised in high yields by modified Biginelli condensation reaction^44^ utilising ethyl cyanoacetate, aromatic aldehyde and thiourea in the presence of potassium carbonate as a base^45–47^ (Scheme 1). Reaction of **4a-i** with 2,3-dichloro-1,4-naphthoquinone **(5)** in DMF afforded the target hybrids, 2-aryl-4,6,11-trioxo-6,11-dihydro-4*H*-naptho[2′,3′:4,5]thiazolo[3,2- *a*]pyrimidine-3-carbonitriles **6a-i** by nucleophilic attack of both NH and SH in compound **4** on both C-Cl in compound **5** under elimination of two hydrogen chloride molecules. The chemical structure of the target **6a-i** hybrids was elucidated utilising the FT-IR, ^1^H NMR, ^13^C NMR and MS spectroscopic data as well as elemental analyses. In the IR spectra of **6a-i** hybrids both significant stretching broad bands due to NH and SH at about 3250 cm^−1^ and 2835 cm^−1^ respectively, were absent. In addition, the band due to the cyano group in compound **4** did not affected by the reaction with **5**. Together with, a sharp intense stretching band in the region 1701–1711 cm^−1^, which advocated the presence of CO group.

In particular, the splitting pattern of the ^1^H NMR spectra of 1,4-naphthoquino group in thiazolopyrimidine derivative **6a**, as an example, exhibited two sets of doublets at δ 7.88–7.48 ppm integrated for two protons, and two sets of triplets at δ 7.36–7.03 ppm for two protons. However, *p-*methoxyphenyl group exhibited two sets of doublets in the range 7.18–6.96 ppm integrated for four protons. The absence of any signals in the region 11.00–13.50 ppm supported the previously route explained for formation of **6a-i** hybrids (Scheme 1). It has been observed that the four protons in the 1,4-naphthoquino group exhibited a multiplet and two doublets in **6c-h,** however only one doublet was detected in **6b** and **6i.** The protons in the *p*-phenylene functionality exhibited, as expected, two doublets in compounds **6a,b,d-,e,h,i,** however in case of **6c,** the presence of two methoxy groups in positions 3 and 4 results in resonating the other three protons as follows: H-2 (s), H-5(d), H-6(d). Moreover, the two equivalent protons in symmetrically 3,4,5-trimethoxyphenyl group in **6f** were detected as a singlet at 6.51 ppm, in addition to two doublets at 7.97 and 7.41 ppm, and one multiplet at 7.87 ppm.

**Figure 4.**
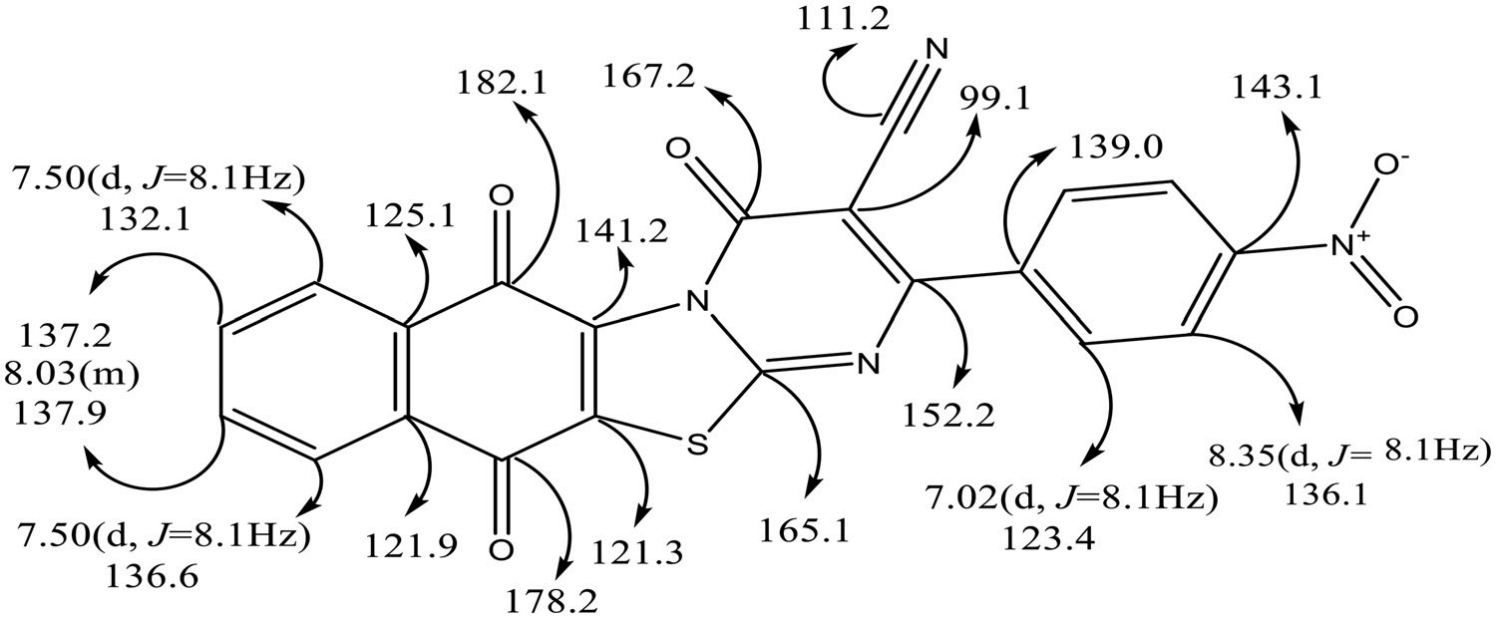
A representative example of ^1^H NMR and ^13^C NMR of **6i** hybrid.

It is worth noting that the symmetry of the un-, mono- as well as tri-substituted aryl group results in decreasing the number of signals in the ^13^C NMR, so that only nineteen signals were observed for the distinctive carbons, in addition to OCH_3_ and CH_3_ carbon atoms. Figure 4 is a representative example for utility of ^1^H and ^13^C NMR spectra in structural assignment of **6i** hybrid.

Additionally, the proposed structures of these compounds were confirmed by mass spectroscopic analyses, where a correct molecular ion peaks with maximum to moderate intensity were detected, which in agreement with the molecular weights of the targeted hybrids **6a-i.** Also, elemental analyses confirmed the elemental compositions of these hybrids.

## Biology

### In vitro cytotoxic activity

*In vitro* cytotoxic activity of the novel synthesised hybrids (**6a-i**) was screened against breast carcinoma (MCF-7), lung carcinoma (A549) and colorectal carcinoma (HCT-116) human cell lines, using MTT assay. Doxorubicin (Dox) and erlotinib were used as reference drugs. The IC_50_ of the synthesised hybrids was evaluated and represented in Table 1. On comparing the IC_50_ values of the titled hybrids, it is clear that most of the synthesised hybrids showed strong to modest cytotoxic activity against the tested human cancer cell lines. In particular, **6i** hybrid experienced the most potent cytotoxic activity among the synthesised hybrids which was evidenced by its IC_50_ values of 1.79 ± 0.11 µM, 1.55 ± 0.13 µM and 2.29 ± 0.18 µM for MCF-7, A549 and HCT-116 cell lines, respectively, that represents 3.13-, 3.19- and 2.96-fold superior activity than Dox and 4.05, 3.35 and 3.77 fold superior activity than erlotinib, respectively. Similarly, **6a** and **6c** hybrids showed potent and efficacious cytotoxic activity greater than that achieved by both Dox and erlotinib which was cleared by their IC_50_ values against the tested cancer cell lines.

**Table 1.** Cytotoxicity of the synthesised hybrids (**6a-i**), dox and erlotinib against MCF-7, A549 and HCT-116 cell lines.

Compound no.	IC_50_ (µM) ± S.E.		
	MCF-7	A549	HCT-116
**6a**	3.34 ± 0.21	2.81 ± 0.18	3.58 ± 0.24
**6b**	4.78 ± 0.36	4.20 ± 0.41	11.54 ± 0.12
**6c**	4.42 ± 0.35	3.65 ± 0.25	3.66 ± 0.39
**6d**	5.66 ± 0.29	3.25 ± 0.31	11.77 ± 0.11
**6e**	5.14 ± 0.32	5.72 ± 0.37	9.81 ± 0.65
**6f**	29.11 ± 1.56	38.27 ± 3.21	26.82 ± 1.78
**6 g**	13.85 ± 1.12	42.17 ± 2.19	9.00 ± 0.66
**6h**	37.94 ± 2.25	21.23 ± 1.23	56.22 ± 4.11
**6i**	1.79 ± 0.11	1.55 ± 0.13	2.29 ± 0.18
**Dox**	5.62 ± 0.31	4.95 ± 0.29	6.78 ± 0.44
**Erlotinib**	7.26 ± 0.2	5.19 ± 0.36	8.64 ± 0.18

Data were expressed as mean ± standard error (SE) of three independent experiments.

Compared to Dox, **6b** and **6d** hybrids displayed superior cytotoxic activity against MCF-7 and A549 cell lines with IC_50_ values of 4.78 ± 0.36 µM and 5.66 ± 0.29 µM for MCF-7 cell line and 4.20 ± 0.41 µM, 3.25 ± 0.31 µM for A549 cell line, respectively. Furthermore, **6b** hybrid experienced potential cytotoxic activity of 1.52- and 1.23-fold the activity of erlotinib against MCF-7 and A549 cell lines, respectively. Similarly, **6d** hybrid achieved 1.28- and 1.60-fold the activity of erlotinib against the same cell lines. On the other hand, **6e** hybrid achieved better cytotoxic activity against MCF-7 cell line with IC_50_ value of 5.14 ± 0.32 µM, compared to Dox. On the other hand, **6f, 6 g** and **6h** hybrids showed weaker cytotoxic activity against the tested cell lines, as indicated by their IC_50_ values, compared to the reference compounds. In conclusion, the preliminary results are encouraging and some of these compounds may serve as potential candidates for development of new anticancer agents.

### Topoisomerase iIα inhibitory activity

**Figure 5.**
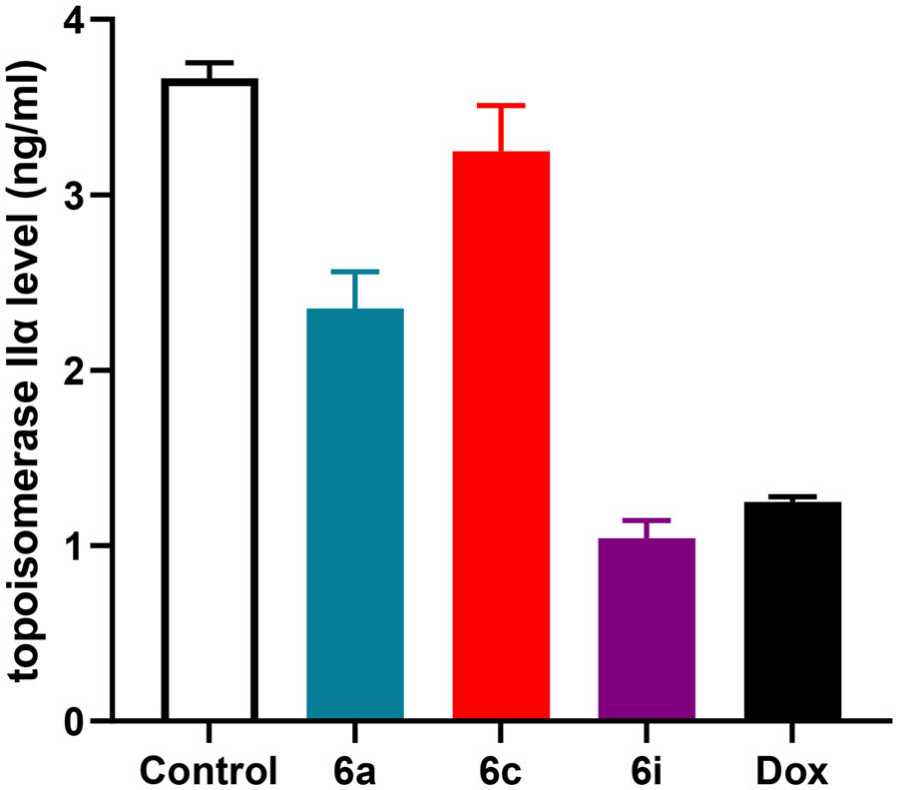
Effect of the tested hybrids (**6a, 6c** and **6i**) and Dox on topoisomerase IIα concentration in MCF-7 cell line.

Out of all the synthesised hybrids, **6a, 6c** and **6i** hybrids that displayed the most potent cytotoxic activity are further investigated to explore their possible mechanism of action as topoisomerase iIα inhibitor. The results were shown in Figure 5 using Dox as a reference compound. It was cleared from the results that **6a, 6c** and **6i** hybrids, at their IC_50_, possessed potent inhibitory activity against topoisomerase iIα. Particularly, **6i** hybrid afforded the highest topoisomerase iIα inhibitory activity, where it decreases the concentration of topoisomerase iIα in MCF-7 cell line to 1.043 ± 0.10 ng/ml, representing 71.50% inhibition of topoisomerase iIα activity compared to the control MCF-7 cell line where the concentration of topoisomerase iIα was 3.663 ± 0.09 ng/ml. Moreover, **6i** hybrid achieved superior topoisomerase iIα inhibitory activity compared to Dox. Dox was able to decrease the concentration of topoisomerase iIα in MCF-7 cell line to 1.248 ± 0.09 ng/ml, representing 65.92% inhibition of topoisomerase iIα activity.

Noteworthy, **6a** hybrid potently reduced topoisomerase iIα concentration to 2.351 ± 0.21 ng/ml that represents 35.81% inhibition of topoisomerase iIα activity compared to control cell. Furthermore, **6c** hybrid exhibited 11.30% inhibition of topoisomerase iIα activity compared to that of MCF-7 cell line. These results support that these hybrids are potent topoisomerase iIα inhibitor; and were in accordance with the *in vitro* cytotoxic activity of these compounds against MCF-7, A549 and HCT-116 cell lines.

### DNA topo II inhibition assay

**Figure 6.**
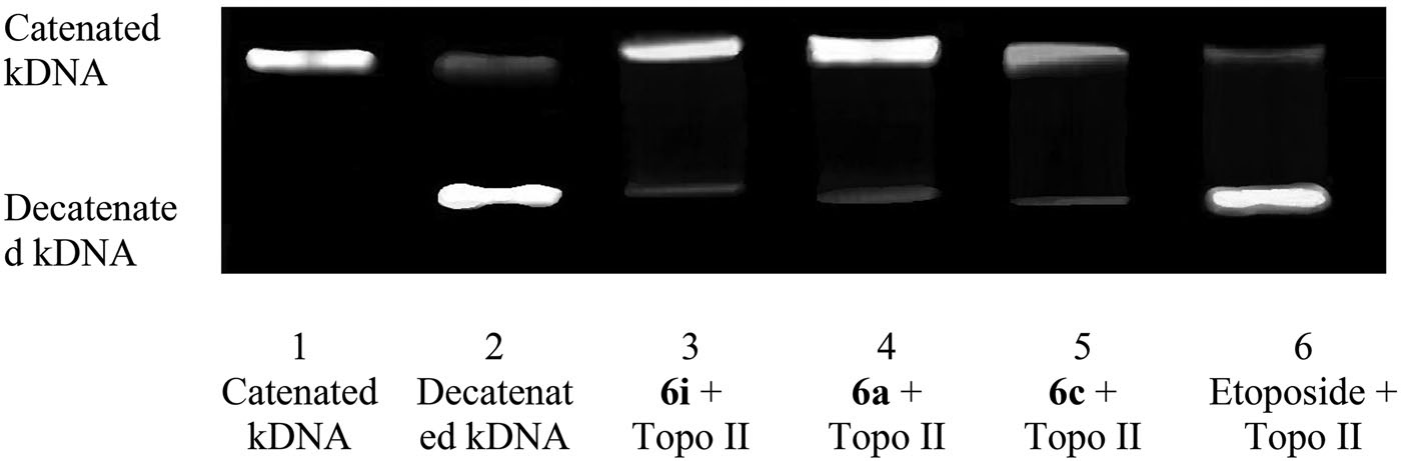
DNA topo II inhibition assay of **6i**, **6a** and **6c**.

In the current assay, the catenated kDNA remained close to the well (lane 1, Figure 6), and after the addition of topo II, the catenated kDNA primarily changed to linear form (lane 2, Figure 6). When the catenated kDNA was incubated with topo II and 10 µM of **6i**, **6a** and **6c** (lanes 3, 4 and 5, respectively, Figure 6), the production of linear DNA was not noticed and the majority of the catenated DNA stayed in the well rather than entering the gel. In conclusion, our compounds may act as catalytic inhibitors of topo II enzyme.

On the other hand, a linear DNA formation was seen when catenated DNA and topo II enzyme were incubated with 10 µM etoposide (Lane 6, Figure 6) indicating that etoposide catalyses DNA breakage and inhibits DNA resealing. This ensures that etoposide serves as topo II poison.

### EGFR-TK inhibitory activity

**Figure 7.**
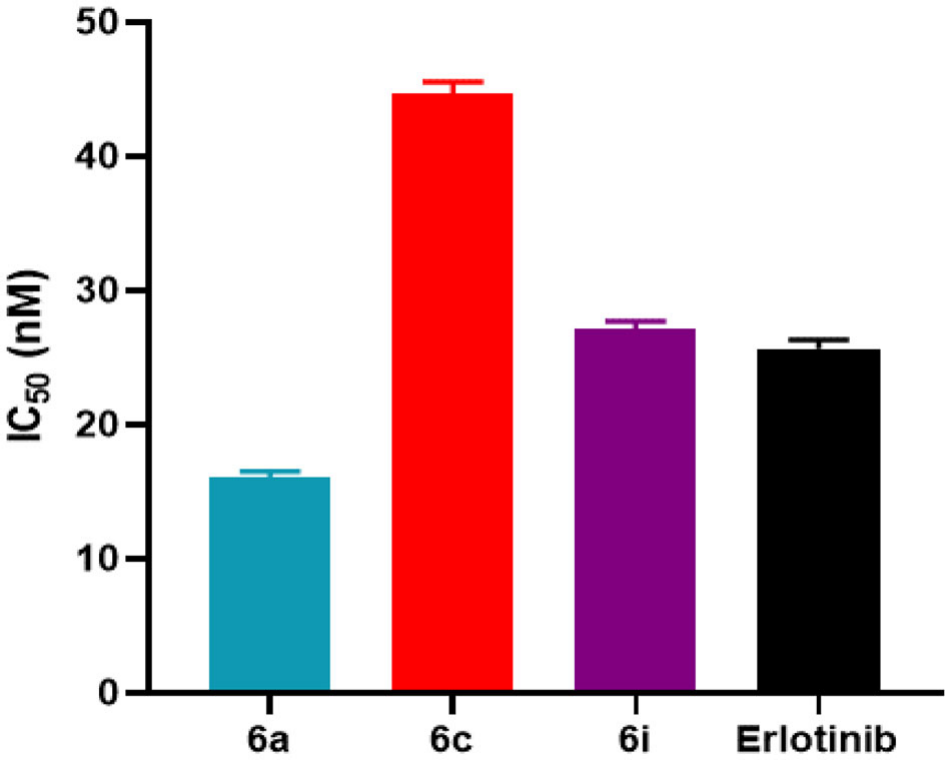
IC_50_ values of the tested hybrids (**6a, 6c** and **6i**) and erlotinib against EGFR.

*In vitro* EGFR inhibitory activity of **6a**, **6c** and **6i** hybrids were assessed and compared to the inhibitory activity of erlotinib that served as reference standard. As shown in Figure 7, **6a** hybrid exhibited EGFR inhibitory activity with IC_50_ values in nanomolar range of 16.03 ± 0.47 nM; while erlotinib showed relatively weaker EGFR inhibitory activity with IC_50_ value of 25.68 ± 0.65 nM. Moreover, **6i** hybrid showed EGFR inhibitory activity nearly equipotent to that of erlotinib. Furthermore, **6c** hybrid achieved lower but remarkable anti-EGFR activity with IC_50_ of 44.72 ± 0.82 nM, compared to erlotinib.

#### Effects of 6a, 6c and 6i hybrids on p53, Bax and Bcl-2 levels in MCF-7 cancer cell line

**Figure 8.**
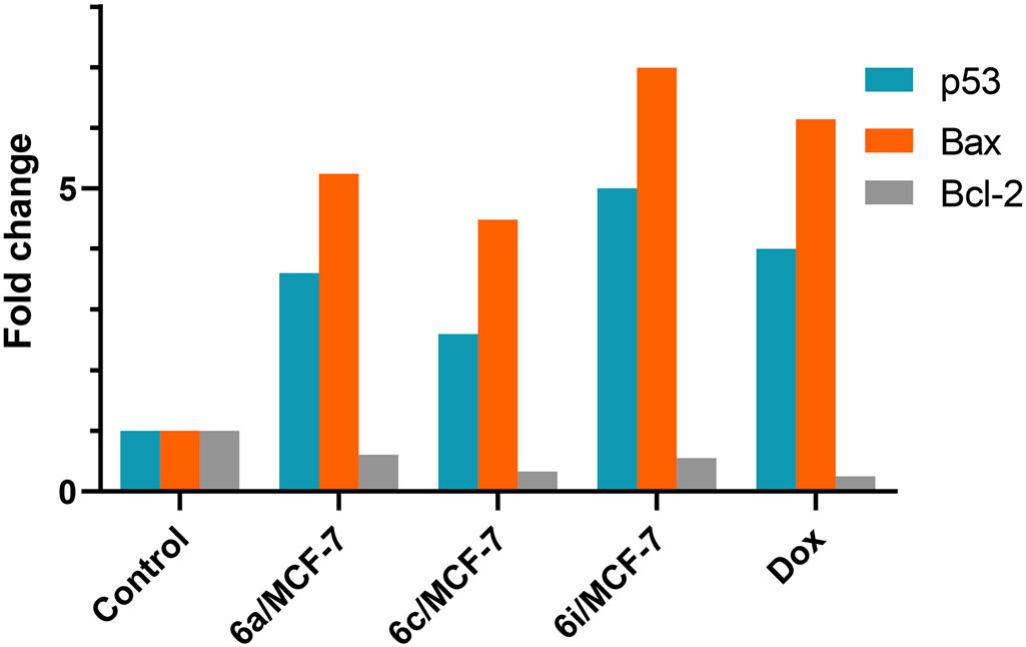
Effects of **6a, 6c** and **6i** hybrids and Dox on p53, Bax and Bcl-2 levels in MCF-7 cancer cell line.

p53 is the major tumour suppressor protein that exerts a fundamental role in preserving the integrity of human genetic information. Reduced p53 activity or mutated p53 gene are implicated in pathogenesis of majority of human cancers. In case of DNA damage or cell stress, p53 induces cell cycle arrest to expand the time required for DNA repair procedures or to induce cell apoptosis^49^. The pro-apoptotic activity of p53 is mediated by regulating the expression of several genes; which have p53 binding sites at their promoter regions. Among these genes, Bcl-2 with anti-apoptotic activity and Bax with pro-apoptotic activity^50^. p53 protein acts as up-stream regulator of Bax. Bax is located in the outer membrane of mitochondria and translocates to mitochondria at the beginning of apoptosis process, proving that it is essential for apoptosis^51^. p53 binds to Bax promotor regions and directly induces its transcription. Bax exists as homodimer or heterodimer complex with Bcl-2^50^. The BAX is deactivated upon heterodimerization with Bcl-2. Bcl-2 family regulates cell survival and prevent cell apoptosis^52^. Bcl-2 up-regulation has been implicate not only in development of many human cancers but also in metastasis and poor prognosis. Importantly, the ratio of individual members of Bcl-2 family is suggested to expose the cell to either suppressed or induced apoptosis upon exposure to certain stimuli^50^. Accordingly, modulation of relative Bax and Bcl-2 levels is fundamental in determining whether cellular apoptosis is initiated or inhibited. Accordingly, the levels of p53, Bax and Bcl-2 were measured in MCF-7 cells after treatment with our most potent cytotoxic compounds. The results were shown in Table 2, Figure 8.

**Table 2.** Effects of **6a**, **6c** and **6i** hybrids on p53, Bax and Bcl-2 levels in MCF-7 cancer cell line.

Compound no.	IC_50_	p53		Bax		Bcl-2	
		Conc.(pg/ml)	FLD	Conc.(pg/ml)	FLD	Conc.(ng/ml)	FLD
**6a**	3.34	922 ± 37.5	3.6	436.1 ± 5.1	5.24	3.99 ± 0.24	0.61
**6c**	4.42	678 ± 42.3	2.6	372.7 ± 9.4	4.48	2.17 ± 0.36	0.33
**6i**	1.79	1285 ± 29.9	5	581.5 ± 8.7	6.99	3.64 ± 0.08	0.55
**Control**	—	257 ± 19.1	1	83.16 ± 6.2	1	6.56 ± 0.22	1
**Dox**	5.62	1051 ± 22.6	4	511.2 ± 6.5	6.14	1.66 ± 0.03	0.25

Data were expressed as mean ± standard error (SE) of three independent experiments.

Furthermore, **6a**, **6c** and **6i** hybrids, at their IC_50_, significantly increased p53 protein level in MCF-7 cell line compared to control cells. Particularly, **6i** hybrid was the most potent inducer of p53 level which was 1285 ± 29.9 pg/ml compared to 257 ± 19.1 pg/ml for control cells and 1051 ± 22.6 pg/ml for Dox treated cells. The increase in p53 level achieved by **6i** hybrid represents 5 fold and 1.2 fold compared to control and Dox treated cells, respectively. Similarly, **6a** and **6c** hybrids achieved p53 level of 922 ± 37.5 pg/ml and 678 ± 42.3 pg/ml, respectively, that represented 3.6-fold and 2.6-fold its concentration in control cells. Additionally, Dox treatment elevated p53 level by 4-fold compared to control cells. Notably, our data are in accordance with the results of apoptosis assays. Simultaneously, **6i** hybrid was able to significantly increase the concentration of pro-apoptotic protein Bax to 581.5 ± 8.7 pg/ml compared to 83.16 ± 6.2 pg/ml for control cells, and on the top of Dox that achieved 511.2 ± 6.5 pg/ml. Moreover, **6a** and **6c** hybrids increased Bax concentration by 5.24 and 4.48 fold, respectively, compared to control cells.

On the other hand, the level of anti-apoptotic protein Bcl-2 was declined to 0.33 and 0.55 its level in control cells after treatment with **6c** and **6i** hybrids, respectively.

#### Effect of 6a, 6c and 6i hybrids on the levels of caspase 7 and 9

At molecular level, apoptosis is regulated via several factors, including the major tumour suppressor protein p53, Bax, Bcl-2, tumour necrosis factor-alpha (TNF-α) as well as caspases family. p53 is involved in regulation of both intrinsic and extrinsic apoptotic pathways^49^. p53 activates the extrinsic pathway by up-regulating TNF-α or Fas receptors with subsequent activation of down-stream signalling cascades that ends by recruitment of effector caspases. Assessment of these caspases serves as efficient indicator for apoptosis. Therefore, in the current study, levels of caspase 7 and 9 were measured to elucidate the effect of our compounds on MCF-7 cells on a molecular level; the results are shown in Table 3.

**Table 3.** Effects of **6a, 6c** and **6i** hybrids on caspase-7 and caspase-9 levels in MCF-7 cancer cell line.

Compound no.	IC_50_	Caspase 7		Caspase 9	
		Conc.(ng/ml)	FLD	Conc.(pg/ml)	FLD
**6a**	3.34	6.72 ± 0.23	14.93	21.17 ± 0.38	4.69
**6c**	4.42	1.89 ± 0.02	4.20	13.68 ± 0.42	3.03
**6i**	1.79	9.20 ± 0.87	20.41	32.29 ± 0.33	5.38
**Control**	—	0.45 ± 0.03	1	4.51 ± 0.29	1
**Dox.**	5.62	10.44 ± 0.92	23.2	29.48 ± 0.25	6.53

Data were expressed as mean ± standard error (SE) of three independent experiments.

Notably, **6i** and **6a** hybrids, at their IC_50_, significantly increased caspase 7 level by 20.41- and 14.93-fold, respectively, compared to control group. Similarly, caspase 9 level was elevated by 5.38 and 4.69-fold after treatment with **6i** and **6a** hybrids, respectively, compared to control cells. In the same context, Dox treatment potently increased caspase 7 and 9 by 23.2- and 6.53-fold, respectively.

### In vitro cell cycle analysis

#### In vitro DNA-flow cytometry

**Figure 9.**
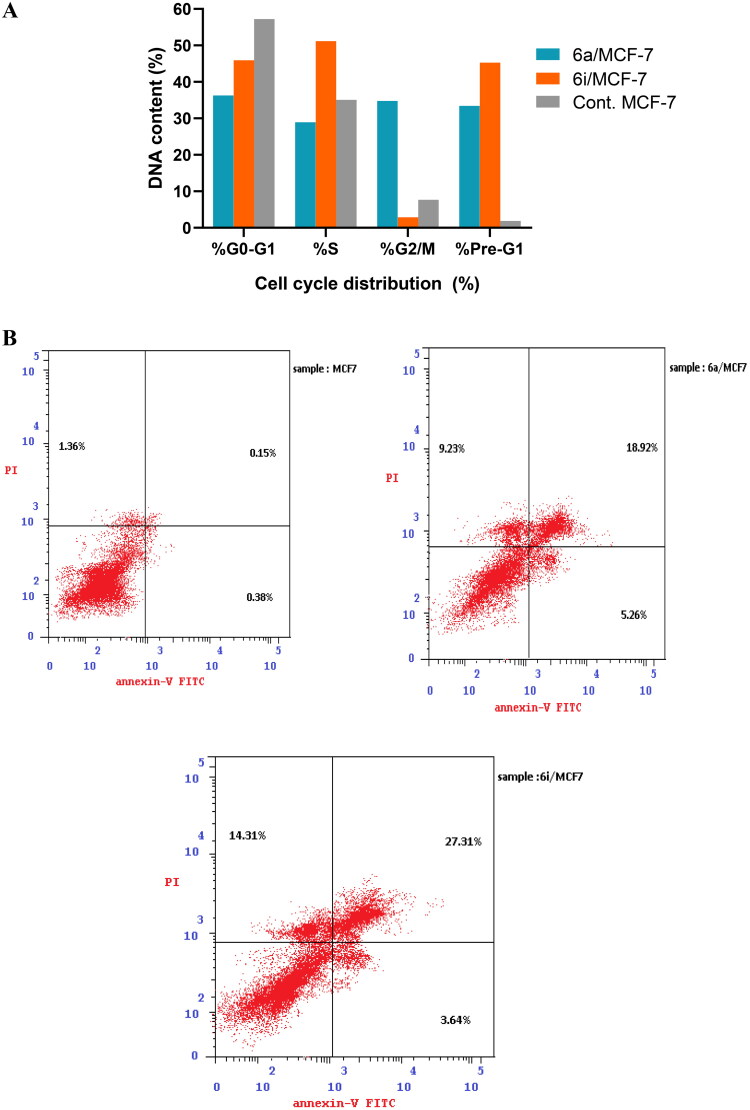
(**A**) Cell cycle analysis in MCF-7 cell line treated with **6a** and **6i** hybrids. (**B**) Cell cycle analysis and apoptosis effect in MCF-7 cell line treated with **6a** and **6i** hybrids.

In order to study the effect of the synthesised hybrids on cell cycle distribution and induction of apoptosis, the most cytotoxic and the most potent topo iIα/EGFR inhibitors **6a** and **6i** hybrids were selected to investigate their effect in MCF-7 cell line using flow cytometer analysis. The results shown in Table 4 and Figure 9(A,B**)** indicated that exposure of MCF-7 cell line to **6a** and **6i** hybrids at their IC_50_ concentration (3.34 µM and 1.79 µM) induced a remarkable disruption in cell cycle profile and cell-cycle arrest.

**Table 4.** Cell cycle analysis of **6a** and **6i** hybrids in MCF-7 cell line.

		DNA content			
Compound no.	IC_50_ µM	%G0-G1	%S	%G2/M	%Pre-G1
**6a**	3.34	36.28	28.94	34.78	33.41
**6i**	1.79	45.94	51.17	2.89	45.26
**Cont.MCF-7**	–	57.23	35.09	7.68	1.89

Furthermore, the results cleared that **6a** and **6i** hybrids decreased cell population significantly in G0/G1 phase, where it was 36.28% and 45.94%, respectively, compared to 57.23% for control MCF-7 cell line. Additionally, the cellular population in S phase was 35.09% in control cell while it decreased to 28.94 upon treating with **6a** hybrid. In G2/M phase, **6a** hybrid was able to cause a significant rise in cell population to reach to 34.78% compared to 7.68% in MCF-7 cell line. Furthermore, pre-G1 phase cell population percentage extremely increased to 33.41% and 45.26% after treatment with **6a** and **6i** hybrids, respectively, compared to control cell (1.89%). Consequently, the results revealed that **6a** and **6i** hybrids significantly arrested the cell cycle in pre-G1phase and G0/G1 phase. Moreover, **6a** hybrid was able to arrest the cell cycle at the G2/M phase and induce apoptosis.

### Annexin V-FITC/PI staining assay

**Figure 10.**
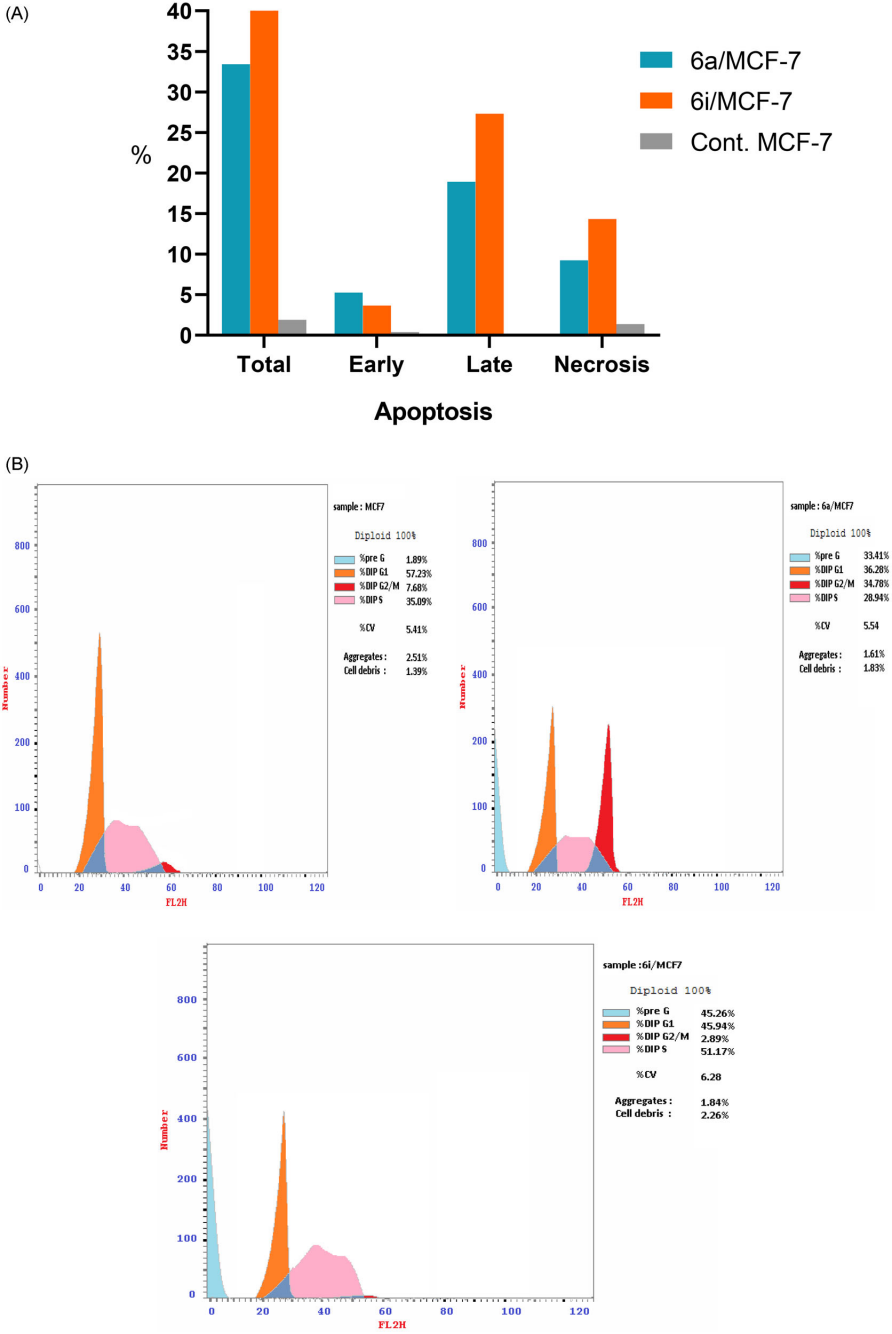
(**A)** Percentage of apoptosis and necrosis for **6a** and **6i** hybrids in MCF-7 cell line. (**B)** Flow cytometric analysis of Annexin V-FITC/PI induced by **6a** and **6i** hybrids in MCF-7 cell line.

The cell cycle results cleared that **6a** and **6i** hybrids exhibited considerable increase in preG1 phase which is a suggestion of apoptosis. Accordingly, to ensure the apoptotic potency and to quantify the apoptosis percentage of the selected hybrids, Annexin V-FITC/propidium iodide dual staining assay was performed in MCF-7 cell line. The results in Table 5 and Figure 10(A,B) showed that the selected hybrids **6a** and **6i** were able to increase cell population in early apoptotic stage by 13.84 and 9.57 folds, respectively, compared to the control cell.

**Table 5.** Results of apoptotic assay of **6a** and **6i** hybrids in MCF-7 cell line.

Compound no.	Apoptosis			Necrosis
	Total	Early	Late	
**6a**	33.41	5.26	18.92	9.23
**6i**	45.26	3.64	27.31	14.31
**Cont.MCF-7**	1.89	0.38	0.15	1.36

On the other hand, the late apoptotic cell population was significantly increased to 126.13 and 182.06 folds upon treatment with **6a** and **6i** hybrids, respectively, compared to the control cell. Meanwhile, **6a** and **6i** hybrids achieved significant increase in cell necrosis by 6.78 and 10.52 folds, respectively, compared to MCF-7 cell line. Finally, these findings evidenced the ability of the selected hybrids for inducing apoptosis at early apoptotic, late apoptotic stages as well as cell necrosis development, which in turn, confirm the ability of the synthesised hybrids to potentially inhibit topo iIα/EGFR activity via the impairment of cell division and apoptosis inducing activity.

## Cheminformatics prediction

### Drug likeliness and oral bioavailability

Computational technology becomes a vital tool in drug candidate identification via contributing in optimising the therapeutic agents design and reducing the experimental drug trials required for drug selection and development. Therefore, physicochemical properties (Lipinskiʼs parameters, topological polar surface area (TPSA)), bioactivity scores and drug-likeness of the synthesised hybrids (**6a-i)** were predicted by using Molinspiration cheminformatics software (http://www.molinspiration.com).

According to lipinskiʼs rule of five, the molecule is considered to be orally active if it obeys the following criteria: (i) lipophilicity or the calculated octanol–water partition coefficient (LogP) ≤ 5, (ii) molecular weight (Mwt) ≤ 500, (iii) number of hydrogen bonds donors (nHBD) ≤ 5, (iv) number of hydrogen bonds acceptors (nHBA) ≤ 10. Moreover, veber’s criteria involving: topological polar surface area (TPSA) < 140 Å and number of rotatable bonds (nRB) < 10. Notably, violation of more than one of these rules indicating bioavailability problems^53^. Gratefully, the results displayed in Table 6 showed that all the synthesised hybrids possessed desirable physicochemical properties with no Lipinski property violations. Noteworthy, Log P of the studied hybrids was in the range of (2.93–4.09) indicating good lipophilicity and permeability across the cell membrane. Additionally, these hybrids can be easily transported and absorbed through the biological membranes where the Mwt was less than 500 Da. Meanwhile, the number of hydrogen bond donors and acceptors were less than 5 and 10, respectively, which was in compliance with Lipinski’s rules.

**Table 6.** Pharmacokinetic prediction of the synthesised hybrids **(6a-i)** by Molinspiration v2021.03.

Comp. no.	miLog P^a^	TPSA^b^	Mwt^c^	nON^d^	nOHNH^e^	N violations^f^	Nrotb^g^	Volume^h^
**6a**	3.34	101.55	413.41	7	0	0	2	331.29
**6b**	3.44	92.31	401.38	6	0	0	1	310.67
**6c**	2.93	110.78	443.44	8	0	0	3	356.83
**6d**	4.09	92.31	462.28	6	0	0	1	323.63
**6e**	3.96	92.31	417.83	6	0	0	1	319.28
**6f**	2.91	120.01	473.47	9	0	0	4	382.38
**6g**	3.28	92.31	383.39	6	0	0	1	305.74
**6h**	3.38	95.55	426.46	7	0	0	2	351.65
**6i**	3.24	138.14	428.38	9	0	0	2	329.08

^a^Logarithm of partition coefficient between n-octanol and water (miLogP).

^b^Topological polar surface area (TPSA)

^c^Molecular weight (Mwt).

^d^Number of hydrogen bond acceptors (n-ON).

^e^Number of hydrogen bond donors (n-OHNH).

^f^Number of violation.

^g^Number of rotatable bonds (N-rotb).

^h^Molecular Volume (Mol.Vol.)

On the other hand, the results cleared that these hybrids also in accordance with Veberʼs rule, where the number of rotatable bonds was less than 10 so these hybrids had been expected to be conformationally stable as well as the TPSA of all the targeted hybrids identified in the range of (92.31–138.14 Å), suggesting good oral bioavailability. Finally, these hybrids were found to be in good agreement with the given criteria and can be said to possess promising drug-like characters with good oral bioavailability.

### Bioactivity score

The bioactivity score is considered a measure of the ability of the potential drug to interact with the different receptors such as G protein-coupled receptor inspection ligand (GPCRL), ion channels, kinases, nuclear receptors, proteases and enzymes. The bioactivity scores of the studied hybrids were calculated by Molinspiration online server where if the bioactivity score is more than 0 the compound is considered to be active; if the bioactivity score is between −5.0 to 0.0 the compound is considered to be moderately active and if the score is less than −5.0 the compound is considered to be inactive [54]. On close inspection of the results tabulated in Table 7, it was cleared that the bioactivity scores of the titled hybrids (**6a-i**) were between −5.0 and 0.0, therefore these hybrids are considered to be moderately active towards the different measured receptors.

**Table 7.** Bioactivity score of the synthesised hybrids (**6a-i**).

Comp. no.	GPCRL	ICM	KI	NRL	PI	EI
**6a**	−0.29	−0.43	−0.04	−0.42	−0.45	−0.20
**6b**	−0.25	−0.39	−0.02	−0.41	−0.44	−0.17
**6c**	−0.28	−0.41	−0.01	−0.42	−0.46	−0.18
**6d**	−0.34	−0.44	−0.04	−0.52	−0.52	−0.22
**6e**	−0.26	−0.38	−0.02	−0.45	−0.45	−0.19
**6f**	−0.28	−0.39	−0.01	−0.46	−0.45	−0.17
**6g**	−0.27	−0.39	−0.00	−0.44	−0.43	−0.16
**6h**	−0.25	−0.38	0.02	−0.39	−0.42	−0.17
**6i**	−0.37	−0.40	−0.12	−0.48	−0.52	−0.24

GPCRL: G protein-coupled receptor Ligand; ICM: Ion channel modulator; KI: Kinase Inhibitor; NRL: Nuclear receptor ligand; PI: Protease inhibitor; EI: Enzyme inhibitor

### In silico ADMET prediction

Pharmacokinetic profile, including absorption, distribution, metabolism, excretion and toxicity, is considered an important parameter that determines the ability of the studied hybrids to act as drug leads. Therefore, pharmacokinetic features of the synthesised hybrids were predicted by using pkCSM pharmacokinetic prediction program.

### Absorption studies

Many important pharmacokinetic parameters have been estimated such as Caco-2 cell permeability, human intestinal absorption (HIA), acting as P-glycoprotein substrate and P-glycoprotein I/II inhibitors. The results tabulated in Table 8 revealed that the synthesised hybrids **(6a-i)** exhibited good water solubility as well as moderate to high Caco-2 cell permeability indicating good *in vivo* absorption rate through the small intestinal wall. Moreover, human intestinal absorption (HIA%) values of all the titled hybrids were in high range from (96.5% to100%) which indicated that these hybrids could be absorbed properly from the GIT upon oral administration. Additionally, the results cleared that these hybrids act as P-glycoprotein I/II inhibitors.

**Table 8.** Absorption properties of the synthesised hybrids (**6a-i**).

Comp.no.	Water solubility	**Caco2** **permeability**	(HIA)%	Skin Permeability	P-glycoprotein substrate	P-glycoprotein I inhibitor	P-glycoprotein II inhibitor
**6a**	−4.336	1.207	100%	−2.723	No	Yes	Yes
**6b**	−4.382	1.227	100%	−2.722	No	Yes	Yes
**6c**	−4.232	0.569	100%	−2.732	No	Yes	Yes
**6d**	−4.718	0.566	100%	−2.716	No	Yes	Yes
**6e**	−4.678	1.123	100%	−2.715	No	Yes	Yes
**6f**	−4.230	0.578	100%	−2.737	No	Yes	Yes
**6g**	−4.618	1.282	99.7%	−2.713	No	Yes	Yes
**6h**	−4.529	0.583	97.3%	−2.715	No	Yes	Yes
**6i**	−4.723	0.434	96.5%	−2.733	No	Yes	Yes

### Distribution studies

The distribution properties provided by pkCSM program are volume distribution (VDss), Fraction unbound (human), blood brain barrier penetration (BBB) and CNS permeability. The results in Table 9 indicated that the synthesised hybrids experienced acceptable VD, unable to penetrate the CNS and consequently poorly distributed to the brain.

**Table 9.** Distribution properties of the synthesised hybrids (**6a-i**).

Comp. no.	VDss	Fraction unbound (human)	BBB permeability	**CNS** **permeability**
**6a**	−0.227	0.275	−1.035	−2.084
**6b**	−0.299	0.290	−1.018	−1.958
**6c**	−0.272	0.270	−1.254	−3.008
**6d**	−0.201	0.266	−0.994	−1.756
**6e**	−0.220	0.267	−0.986	−1.779
**6f**	−0.422	0.253	−1.477	−3.188
**6g**	−0.291	0.255	−0.798	−1.888
**6h**	−0.155	0.267	−0.956	−1.984
**6i**	−0.396	0.236	−1.316	−2.097

### Metabolism and excretion studies

The results in Table 10 cleared that all the synthesised hybrids predicted to be substrate for CYP3A4 and inhibitors for CYP1A2, CYP2C19, CYP2C9 isoforms except for **6f** hybrid. Moreover, they are neither substrate for CYP2D6 or inhibitor for CYP2D6 and CYP3A4 isoforms.

**Table 10.** Metabolism and excretion properties of the synthesised hybrids (**6a-i**).

Comp. no.	CYP2D6 substrate	CYP3A4 substrate	CYP1A2 inhibitior	CYP2C19 inhibitior	CYP2C9 inhibitior	CYP2D6 inhibitior	CYP3A4 inhibitior	Renal OCT2 Substrate
**6a**	No	Yes	Yes	Yes	Yes	No	No	No
**6b**	No	Yes	Yes	Yes	Yes	No	No	No
**6c**	No	Yes	Yes	Yes	Yes	No	No	No
**6d**	No	Yes	Yes	Yes	Yes	No	No	No
**6e**	No	Yes	Yes	Yes	Yes	No	No	No
**6f**	No	Yes	No	No	No	No	No	No
**6g**	No	Yes	Yes	Yes	Yes	No	No	No
**6h**	No	Yes	Yes	Yes	Yes	No	No	No
**6i**	No	Yes	Yes	Yes	Yes	No	No	No

### Toxicity studies

The results obtained from Table 11 displayed that the estimated maximum tolerated dose for human was higher than that of Dox. Notably, the inhibition of human ether-a-go-go-related gene (hERG) cardiac potassium channel is considered a crucial factor in inhibiting cardiac arrhythmias. All the synthesised hybrids (**6a-i**) predicted to show inhibitory activity against hERG I and hERG II (except for **6a, 6c** and **6f** hybrids). Meanwhile, LD_50_ value for all tested hybrids was comparable to that of Dox On the other hand, all the studied hybrids (**6a-i**) exerted no hepatotoxic potential, except for **6b** and **6f** hybrids. These findings strongly confirm the ability of most of the titled hybrids to act as a drug.

**Table 11.** Toxicity properties of the synthesised hybrids (**6a-i**) and Dox.

Comp. No.	Max. tolerated dose (human)	hERG I inhibitor	hERG II inhibitor	Oral Rat Acute Toxicity (LD_50_)	Hepatotoxicity
**6a**	0.337	No	Yes	2.523	No
**6b**	0.353	No	No	2.467	Yes
**6c**	0.366	No	Yes	2.653	No
**6d**	0.292	No	No	2.373	No
**6e**	0.289	No	No	2.368	No
**6f**	0.427	No	Yes	2.748	Yes
**6g**	0.424	No	No	2.322	No
**6h**	0.288	No	No	2.441	No
**6i**	0.207	No	No	2.980	No
**Dox.**	0.115	No	No	2.408	No

## Molecular docking study

### Docking at topo iIα ATPase active site

**Figure 11.**
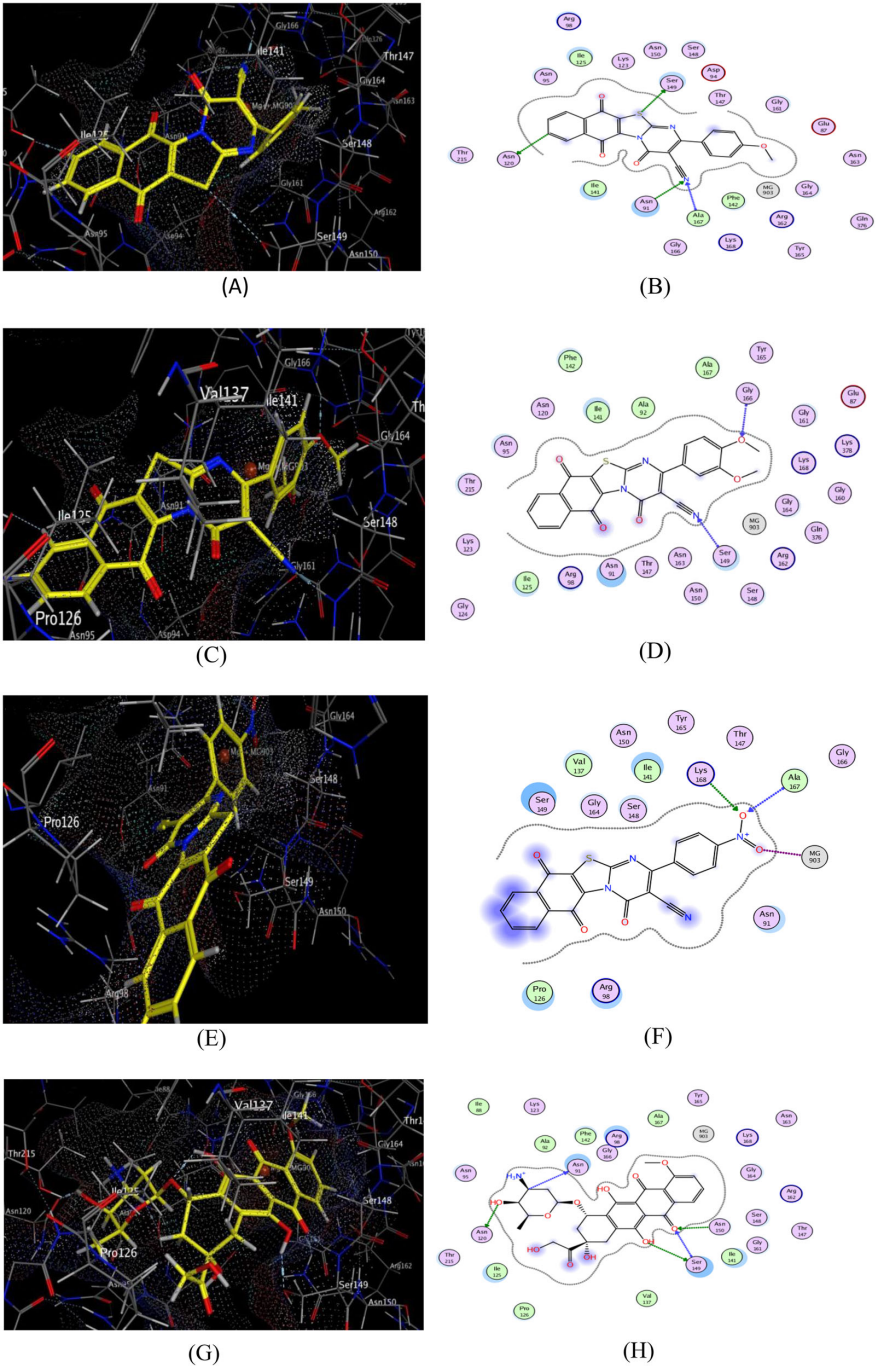
Docking and binding pattern of hybrids **6a** (**A**&**B**), **6c** (**C**&**D**), **6i** (**E**&**F**) and Dox. (**G**&**H**) showing interactions with different amino acid residues found in the active site of human topo IIα ATPase (PDB code 1ZXM).

To shed light on the potential binding modes and the crucial binding features of the synthesised hybrids (**6a-i**) with ATPase domain of human topo iIα, molecular docking study was performed using X-ray crystallographic structure of the human topo iIα ATPase/AMP-PNP was obtained from protein databank [PDB code 1ZXM, resolution 1.87 Å (www.rcsb.org)]. Dox was used as a reference topo iIα inhibitor. The results of the binding interactions and the energy binding scores are depicted in Table 12 and Figure 11. Notably, studying T-shaped cavity of ATP binding domain found that it contains three important binding pockets: pocket I composed of various key amino acids including Asn150, Ser148, Ser149, Lys157 and Tyr34. While, pocket II contains essential amino acids including Asn91 and Asn120. Additionally, pocket III contains crucial amino acids such as Ala167, Gly166, Lys168, Tyr165, and Thr147 in addition to Mg^2+^ ion^14^. Noteworthy, it was reported that the interaction with Mg^2+^ ion has been considered essential for the catalytic activity at the ATPase binding domain^55^.

**Table 12.** Types of binding interactions and energy scores (kcal/mol) for hybrids (**6a–i)** and Dox at the human topo IIα ATPase/AMP-PNP active site.

Compound No.	Types of interactions	Energy scores
**6a**	- One hydrogen bonding interaction with Asn 120 (3.30 Å). - One hydrogen bonding interaction with Ser 149 (3.35 Å). - One hydrogen bonding interaction with Asn 91 (3.21 Å). - One hydrogen bonding interaction with Ala 167 (3.18 Å).	−8.16
**6b**	- One hydrogen bonding interaction with Ser 149 (3.19 Å).	−7.52
**6c**	- One hydrogen bonding interaction with Ser 149 (2.79 Å). - One hydrogen bonding interaction with Gly 166 (2.47 Å).	−8.42
**6d**	- One hydrogen bonding interaction with Arg 98 (3.41 Å).	−6.39
**6e**	- One hydrogen bonding interaction with Lys 157 (3.01 Å). - One hydrogen bonding interaction with Thr 215 (2.89 Å).	−7.86
**6f**	- One hydrogen bonding interaction with Arg 98 (3.05 Å). - One π-hydrogen bonding interaction with Ser 148 (4.64 Å).	−5.58
**6g**	- One hydrogen bonding interaction with Asn 120 (3.17 Å).	−7.01
**6h**	- One hydrogen bonding interaction with Thr 215 (3.44 Å).	−5.84
**6i**	- One hydrogen bonding interaction with Ala 167 (3.35 Å). - One hydrogen bonding interaction with Lys 168 (3.09 Å). - One metal-ion interaction with Mg^+2^ (903) (2.47 Å).	−9.51
**Dox.**	- Two hydrogen bonding interaction with Ser 149 (2.57 and 3.12 Å). - One hydrogen bonding interaction with Asn 91 (3.32 Å). - One hydrogen bonding interaction with Asn 120 (2.47 Å). - One hydrogen bonding interaction with Asn 150 (2.58 Å).	−8.35

In close inspection of the results in Table 12 revealed the ability of nearly all of the tested hybrids to interact effectively with the key amino acids in the ATPase domain of human topoIIα compared to that of Dox. Interestingly, **6i** hybrid which possessed the highest topo iIα inhibitory activity, showed efficient binding pattern with the key amino acid residues in the target protein and consequently the highest energy binding score, where it was −9.51 kcal/mol higher than that of the reference compound. Moreover, **6i** hybrid was able to interact efficiently with pocket III through the formation of two hydrogen bonds between one oxygen of the free nitro group with Ala167 and Lys168. Additionally, the another oxygen of the free nitro group was able to form metal-ion interaction with Mg^2+^ ion which considered one of the crucial interactions that underlying the mechanism of topoIIα inhibition where, the entry of ATP into the binding site is hindered by the interaction of the compound with the Mg^2+^ ion^14^. Together with another hydrophobic interaction with Ser 149, Arg 98, Asn 91, Pro 126 and Ile 141 (Figure 11(E,F)).

Of considerable interest, **6a** hybrid was engaged in a network of four H-bonds with the key amino acids in the three pockets of the target protein which have a vital role in the enhancement of topoIIα inhibitory activity. Moreover, this hybrid showed a promising binding mode as S- atom bind *via* H-bond with Ser 149 in pocket I, as well as, the nitrogen of the cyano group and naphthoquinone phenyl ring participated with two H-bonds with Asn 120 and Asn 91in pocket II, together with another H-bond formed between C≡N group and Ala 167 in pocket III (Figure 11(A,B)). On the other hand, the methoxyl oxygen and the nitrogen of the cyano group of **6c** hybrid showed good fitting with pockets I and III *via* formation of two strong H-bonds with Ser 149 and Gly 166, respectively. Additionally, there is an important hydrophobic interaction with Asn 91 (Figure 11(C,D)).

Furthermore, the electron-withdrawing atoms in hybrids **6b, 6d** and **6e** caused slight decrease in the cytotoxic activity compared to the previous mentioned hybrids but they still potent enough for inhibiting the enzyme activity as their IC_50_ values were nearly equipotent to that of Dox where, they able to interact with pocket I through formation of three hydrogen bonds with Ser 149, Arg 98 and Lys 157, respectively. Meanwhile, the steric factor resulting from the large size of the trimethoxy group in **6f** and the dimethyl amino group in **6h** hybrids negatively affects their binding pattern with the receptor site and hence their cytotoxic activity as they could not be able to bind with the crucial amino acids responsible for the activity. Finally, it can be concluded that most of the titled synthesised hybrids exhibited favourable binding interactions with topo iIα target protein in distinct manner and thus are able to inhibit its activity efficiently with pronounced cytotoxic activity.

### Docking at EGFR active site

In order to predict the promising inhibitory activities of the newly synthesised hybrids against ATP active sites of EGFR kinase, the 3D crystal structure (PDB ID: 1XKK) was used in this study and erlotinib was used as a reference compound. Importantly, it was found that the key amino acid residues in EGFR kinase protein were Met 793, ASP 800, Cys797, Asp 855, Arg 841, Thr854, Asp831, Leu718, Gly721, Thr790, Phe 856 and Gly 796^56–59^. It was cleared from the results of energy binding scores and binding interactions in Table 13 that most of the synthesised hybrids bind efficiently with the key amino acids residues in the ATP active sites of EGFR kinase.

**Table 13.** Types of binding interactions and energy scores (kcal/mol) for hybrids (**6a–i)** and erlotinib at the EGFR kinase active site.

Compound no.	Types of interactions	Energy scores
**6a**	- One hydrogen bonding interaction with Met 793 (3.61 Å). - Two hydrogen bonding interactions with Asp 855 (3.62 and 2.92 Å). - One hydrogen bonding interaction with Lys 745 (3.16 Å). - Two π-hydrogen bonding interaction with Val 726 (3.98 and 4.42 Å).	−8.72
**6b**	- One hydrogen bonding interaction with Asp 855 (3.28 Å). - Two π-hydrogen bonding interaction with Val 726 (3.96 and 4.01 Å).	−6.41
**6c**	- One hydrogen bonding interaction with Asp 855 (3.51 Å). - One hydrogen bonding interaction with Lys 745 (3.60 Å). - One π-hydrogen bonding interaction with Val 726 (4.03 Å).	−7.32
**6d**	- One π-hydrogen bonding interaction with Leu 844 (3.86 Å).	−5.89
**6e**	- One π-hydrogen bonding interaction with Leu 844 (3.86 Å).	−5.78
**6f**	- One π-hydrogen bonding interaction with Leu 718 (4.56 Å). - Two π-hydrogen bonding interaction with Val 726 (4.41 and 4.00 Å).	−4.95
**6 g**	- Two π-hydrogen bonding interaction with Val 726 (4.44 and 4.05 Å).	−6.01
**6h**	- One hydrogen bonding interaction with Lys 745 (3.10 Å). - One π-hydrogen bonding interaction with Leu 718 (4.58 Å).	−6.67
**6i**	- One hydrogen bonding interaction with Met 793 (3.66 Å). - Two hydrogen bonding interactions with Asp 855 (2.91 and 3.61 Å). - One hydrogen bonding interaction with Lys 745 (3.17 Å). - Two π-hydrogen bonding interaction with Val 726 (3.98 and 4.41 Å).	−8.25
**Erlotinib**	- One hydrogen bonding interaction with Met 793 (3.48 Å). - Two π-hydrogen bonding interaction with Leu 718 and Thr 854, Thr 790, Gln 791 through H_2_O (4) mediated bond (3.94 Å and 3.79 Å). - One hydrogen bonding interaction with H_2_O (22) (2.93 Å)	−7.33

**Figure 12.**
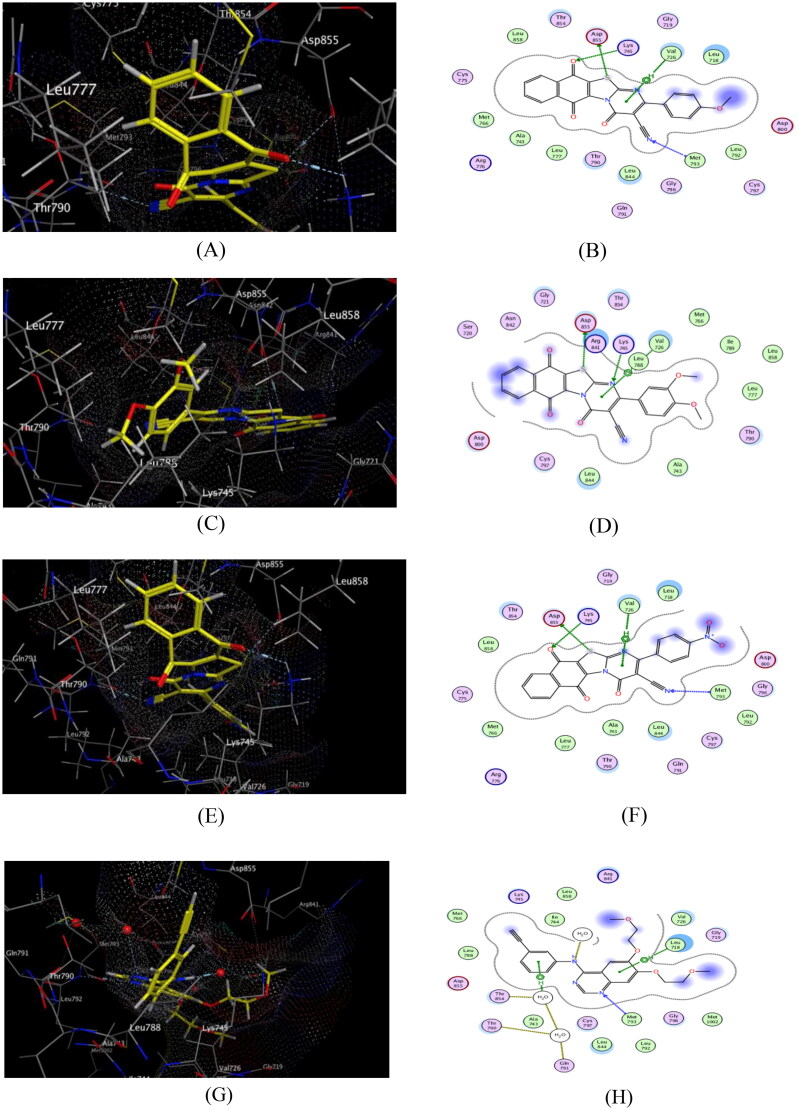
Docking and binding pattern of compounds **6a** (**A**&**B**), **6c** (**C**&**D**), **6i** (**E**&**F**) and erlotinib (**G**&**H**) showing interactions with different amino acid residues found in the ATR-active site of EGFR (PDB code: 1XKK).

Of considerable interest, **6a** and **6i** hybrids achieved better interactions with EGFR-ATP active site where the binding energy scores were higher than that of erlotinib which explained the highest experimental EGFR inhibitory activity. Additionally, the nitrogen of C≡N in both **6a** and **6i** hybrids interacts *via* hydrogen bonding with the key amino acid Met 793. Additionally, these hybrids participated in three hydrogen bonding interactions with Asp 855 and Lys 745 besides two π-H interactions with the key amino acid Val 726 (Figure 12). Moreover, some hydrophobic interactions is formed with important amino acids such as Leu 718, Val 726 (for **6a** hybrid) and Leu 718, Val 726, Leu 844 (for **6c** hybrid). Furthermore, S and N-atoms of thiazolopyrimidine ring of **6c** hybrid formed two hydrogen bonds with Asp 855 and Lys 745 in addition to π-H interaction with Val 726 and hydrophobic interactions with Arg 841, Val 726, Leu 844, Gly 721 and Thr 790 (Figure 12).

Similarly, hydrogen bond is formed between S-atom of thiazolopyrimidine ring of **6b** hybrid and Asp 855, besides two π-H interactions was formed with Val 726. However, the dimethylamino **6h** hybrid formed one hydrogen bonding interaction with Lys 745 together with another π-H interactions with Leu 718. Meanwhile, the electron-withdrawing (**6d** and **6e** hybrids), the trimethoxy **6f** hybrid and the unsubstituted **6 g** hybrid formed weak π-H interactions with Leu 844, Leu 718 and Val 726, respectively which was in accordance with their weak cytotoxic activity.

## Structure–activity relationship

Based on the aforementioned results, it becomes obvious that the nature of the different substituents on the phenyl ring greatly influence the topoIIα/EGFR inhibitory activity and consequently the cytotoxic activity of the studied hybrids. Interestingly, **6i** hybrid experienced the highest topoIIα/EGFR inhibitory activity and in turn, the highest cytotoxic activity owing to the unique ionic nature of the nitro group which was also evidenced by its higher energy binding score and its favourable binding interactions. Furthermore, the lipophilic electron donating methoxy group of **6a** hybrid and dimethoxy group of **6c** hybrid reflected on the efficient binding with the key amino acid residues in the receptor site resulting in good topoIIα/EGFR inhibitory activity and consequently remarkable cytotoxic activity.

Meanwhile, the hydrophilic electron-withdrawing atoms in **6b**, **6d** and **6e** hybrids seemed to slightly decrease their cytotoxic activity compared to **6a** and **6i** hybrids but they still potent enough compared to IC_50_ value of Dox and erlotinib which could be contributed to their ability to form hydrogen bonds with the essential amino acids of the target protein. On the contrary, the steric factor of the dimethyl amino group in **6h** hybrid and the trimethoxy group of **6f** hybrid hinder their proper fitting to the receptor active site and negatively affected their cytotoxic activity which was evidenced by their IC_50_ values. Finally, it is possible to conclude that the electronic nature, lipophilicity, and steric factor of the substituents all have a significant impact on the biological activity of the system incorporated.

## Conclusion

A novel series of naphtho[2′,3′:4,5]thiazolo- [3,2-*a*]pyrimidine hybrids have been designed and synthesised as potential topo II/EGFR inhibitors. Moreover, the cytotoxic activity of the synthesised hybrids was evaluated against MCF-7, A549 and HCT-116 cell lines using Dox and erlotinib as reference compounds using MTT assay. Among the synthesised hybrids, **6i**, **6a** and **6c** hybrids possessed superior anticancer activity of IC_50_ values less than that of the reference drugs. Additionally, **6i** and **6a** hybrids efficiently inhibited topo iIα activity by 71.50% and 35.81%, respectively. Moreover, according to DNA topo II inhibition assay, our compounds may act as catalytic inhibitors of topo II enzyme. Furthermore, these hybrids experienced good EGFR inhibitory activity compared to erlotinib. Meanwhile, these hybrids displayed strong apoptotic activity as evident by accumulation of cells at pre-G1phase and G0/G1 phase, as well as **6a** hybrid was able to arrest the cell cycle at G2/M phase and induce apoptosis. The apoptotic inducer activity of these hybrids was further confirmed by the remarkable rise in p53 level simultaneously with elevation of Bax/Bcl-2 ratio, caspase-7 and caspase-9. Importantly, the synthesised hybrids have drug likeness characters and the molecular docking study revealed that most of the synthesised hybrids possessed appropriate binding pattern with topo iIα and EGFR active sites.

## References

[1] Thakor AS, Gambhir SS. Nanooncology: the future of cancer diagnosis and therapy. CA Cancer J Clin. 2013;63(6):395–418. 24114523 10.3322/caac.21199

[2] Petrelli A, Giordano S. From single- to multi-target drugs in cancer therapy: when a specificity becomes an advantage. Curr. Med. Chem. 2008; 15:422–432. 18288997 10.2174/092986708783503212

[3] Nepali K, Sharma S, Sharma M, Bedi PMS, Dhar KL. Rational approaches, design strategies, structure activity relationship and mechanistic insights for anticancer hybrids. Eur J Med Chem. 2014;77:422–487. 24685980 10.1016/j.ejmech.2014.03.018

[4] Justine L, Hsieh D, Chan N, Hiasa H. Topoisomerases as anticancer targets. Biochem. J. 2018;47:373–398. 10.1042/BCJ20160583 PMC6110615 29363591

[5] Forterre P, Gribaldo S, Gadelle D, Serre MC. Origin and evolution of DNA topoisomerases. Biochimie. 2007;89(4):427–446. 17293019 10.1016/j.biochi.2006.12.009

[6] Nitiss JL. Targeting DNA topoisomerase II in cancer chemotherapy. Nat Rev Cancer. 2009;9(5):338–350. 19377506 10.1038/nrc2607 PMC2748742

[7] Nandi S, Saxena AK. Exploring targets of cell wall protein synthesis and overexpression mediated drug resistance for the discovery of potential M. tb inhibitors. Curr Top Med Chem. 2021;21(21):1922–1942. 34315374 10.2174/1568026621666210727165742

[8] Champoux JJ. DNA topoisomerases: structure, function, and mechanism. Annu Rev Biochem. 2001;70:369–413. 11395412 10.1146/annurev.biochem.70.1.369

[9] McClendon AK, Osheroff N. DNA topoisomerase II, genotoxicity, and cancer, Mutat. Mutat Res. 2007;623(1-2):83–97. 17681352 10.1016/j.mrfmmm.2007.06.009 PMC2679583

[10] Mueller-Planitz F, Herschlag D. DNA topoisomerase II selects DNA cleavage sites based on reactivity rather than binding affinity. Nucleic Acids Res. 2007;35(11):3764–3773. 17517767 10.1093/nar/gkm335 PMC1920260

[11] Bates A, Berger J, Maxwell A. The ancestral role of ATP hydrolysis in type II topoisomerases: prevention of DNA double-strand breaks. Nucleic Acids Res. 2011;39(15):6327–6339. 21525132 10.1093/nar/gkr258 PMC3159449

[12] Linka R, Porter A, Volkov A, Mielke C, Boege F, Christensen M. C-terminal regions of topoisomerase II alpha and II beta determine isoform-specific functioning of the enzymes in vivo. Nucleic Acids Res. 2007;35(11):3810–3822. 17526531 10.1093/nar/gkm102 PMC1920234

[13] Stuart K. Calderwood. A critical role for topoisomerase iIb and DNA double strand breaks in transcription. Transcription. 2016;7(3):75–83. 27100743 10.1080/21541264.2016.1181142 PMC4984685

[14] Hu W, Huang X-S, Wu J-F, Yang L, Zheng Y-T, Shen Y-M, Li Z-Y, Li X. Discovery of Novel Topoisomerase II Inhibitors by Medicinal Chemistry Approaches. J Med Chem. 2018;61(20):8947–8980. 29870668 10.1021/acs.jmedchem.7b01202

[15] Chen SF, Huang NL, Lin JH, Wu CC, Wang YR, Yu YJ, Gilson MK, Chan NL. Structural insights into the gating of DNA passage by the topoisomerase II DNA-gate. Nat Commun. 2018;9(1):3085. 30082834 10.1038/s41467-018-05406-y PMC6078968

[16] Montecucco A, Zanetta F, Biamonti G. Molecular mechanisms of etoposide. Excli J. 2015;14:95–108. 26600742 10.17179/excli2015-561 PMC4652635

[17] Farsani MR, Ganjalikhany SV. Mitoxantrone. More than Just Another Topoisomerase II Poison. Curr Cancer Drug Targets. 2017;17:657–668. 27834128 10.2174/1568009617666161109142629

[18] Yanhe L, Chen G, Ronald S, James H, et al. Humanin analog enhances the protective effect of dexrazoxane against doxorubicin-induced cardiotoxicity. Am J Physiol Heart Circ Physiol. 2018;315:634–643. 10.1152/ajpheart.00155.2018 PMC6734085 29775411

[19] Chen SH, Chan NL, Hsieh TS. New Mechanistic and Functional Insights into DNA Topoisomerases. Annu Rev Biochem. 2013;82:139–170. 23495937 10.1146/annurev-biochem-061809-100002

[20] Vos SM, Tretter EM, Schmidt BH, Berger JM. All tangled up: how cells direct, manage and exploit topoisomerase function. Nat Rev Mol Cell Biol. 2011;12(12):827–841. 22108601 10.1038/nrm3228 PMC4351964

[21] Pogorelčnik B, Perdih A, Solmajer T. Recent advances in the development of catalytic inhibitors of human DNA topoisomerase iIa as novel anticancer agents. Curr Med Chem. 2013;20(5):694–709. 23210851 10.2174/092986713804999402

[22] Sabbah A, Hajjo R, Sweidan K. Review on epidermal growth factor re-odellr (EGFR) structure, signaling pathways, interactions, and recent updates of EGFR inhibitors. Curr Top Med Chem. 2020;20(10):815–834. 32124699 10.2174/1568026620666200303123102

[23] Nandi S, Dey R, Samadder A, Saxena A, Saxena AK. Natural sourced inhibitors of EGFR, PDGFR, FGFR and VEGFR mediated signaling pathways as potential anticancer agents. Curr Med Chem. 2022;29(2):212–234. 33655823 10.2174/0929867328666210303101345

[24] Stewart EL, Tan SZ, Liu G, Tsao M. Known and putative mechanisms of resistance to EGFR targeted therapies in NSCLC patients with EGFR mutations-a review. Transl. Lung Cancer Res. 2015;4:67–81. 25806347 10.3978/j.issn.2218-6751.2014.11.06 PMC4367712

[25] Cohen MH, Johnson JR, Chen YF, Sridhara R, Pazdur R. FDA drug approval summary: erlotinib (TarcevI)) tablets. Oncologist. 2005;10(7):461–466. 16079312 10.1634/theoncologist.10-7-461

[26] Roskoski R. FDA-approved small molecule protein kinase inhibitors. Pharmacol Res. 2019;144:19–50. 30877063 10.1016/j.phrs.2019.03.006

[27] Cai D, Zhang Z-H, Chen Y, Yan X-J, Zhang S-T, Zou L-J, Meng L-H, Li F, Fu B-J. Synthesis of some new thiazolo[3,2-a]pyrimidine derivatives and screening of their *in vitro* antibacterial and antitubercular activities. Med Chem Res. 2016;25(2):292–302.

[28] Khalilpour A, Asghari S, Pourshab M. Synthesis and characterization of novel thiazolo[3,2-a]pyrimidine derivatives and evaluation of antioxidant and cytotoxic activities. codellingvers. 2019;16(5):1800563. 10.1002/cbdv.201800563 30740903

[29] Banothu J, Khanapur M, Basavoju S, Bavantula R, Narra M, Abbagani S. Synthesis, characterization and biological evaluation of fused thiazolo[3,2-a]pyrimidine derivatives. RSC Adv. 2014;4(44):22866–22874.

[30] Abdel Moty SG, Hussein MA, Abdel Aziz SA, Abou-Salim AM. Design and synthesis of some substituted thiazolo[3,2-a]pyrimidine derivatives of potential biological activities. Saudi Pharm J. 2016;24(2):119–132. 27013904 10.1016/j.jsps.2013.12.016 PMC4792894

[31] Tozkoparan B, Ertan M, Kelicen P, Demirdamar R. Synthesis and anti-inflammatory activities of some thiazolo[3,2-a]pyrimidine derivatives. Farmaco. 1999;54(9):588–593. 10555260 10.1016/s0014-827x(99)00068-3

[32] Mohamed SF, Abbas MH, Khalaf HS, Farghaly TA, Abd El-Shafy DN. Triazolopyrimidines and thiazolopyrimidines: synthesis, anti-HSV-1, cytotoxicity and mechanism of action. Mini Rev Med Chem. 2018;18(9):794–802. 29219053 10.2174/1389557518666171207161542

[33] Sekhar T, Thriveni P, Venkateswarlu A, Daveedu T, Peddanna K, Sainath SB. One-pot synthesis of thiazolo[3,2-a]pyrimidine derivatives, their cytotoxic evaluation and molecular docking studies. Spectrochim Acta A Mol Biomol Spectrosc. 2020;231:118056–118064. 32006911 10.1016/j.saa.2020.118056

[34] Hassan GS, El-Messery SM, Abbas A. Synthesis and anticancer activity of new thiazolo[3,2-a]pyrimidines: DNA binding and molecuodellingling study. Bioorg Chem. 2017;74:41–52. 28750204 10.1016/j.bioorg.2017.07.008

[35] Nemr MT, AboulMagd AM. New fused pyrimidine derivatives with anticancer activity: Synthesis, topoisomerase II inhibition, apoptotic inducing activity and molecuodellingling study. Bioorg Chem. 2020;103:104134. 32750610 10.1016/j.bioorg.2020.104134

[36] Gupta AK, Bhunia SS, Balaramnavar VM, Saxena AK. Pharmacophore modelling, molecular docking and virtual screening for EGFR (HER 1) tyrosine kinase inhibitors. SAR QSAR Environ Res. 2011;22(3):239–263. 21400356 10.1080/1062936X.2010.548830

[37] Lin R, Johnson SG, Connolly PJ, Wetter SK, Binnun E, Hughes TV, Murray WV, Pandey NB, Moreno-Mazza SJ, Adams M, et al. Synthesis and evaluation of 2,7-diamino-thiazolo[4,5-d]pyrimidine analogues as anti-tumor epidermal growth factor receptor (EGFR) tyrosine kinase inhibitors. Bioorg Med Chem Lett. 2009;19(8):2333–2337.,. 19286381 10.1016/j.bmcl.2009.02.067

[38] Fahmy HTY, Rostom SAF, Saudi MN, Zjawiony JK, Robins DJ. Synthesis and *in vitro* evaluation of the anticancer activity of novel fluorinated thiazolo[4,5-d] pyrimidines. Arch Pharm (Weinheim)). 2003;336(4-5):216–225. 12916055 10.1002/ardp.200300734

[39] Kumar BS, Ravi K, Verma AK, Fatima K, Hasanain M, Singh A, Sarkar J, Luqman S, Chanda D, Negi AS, et al. Synthesis of pharmacologically important naphthoquinones and anticancer activity of 2-benzyllawsone through DNA topoisomerase-II inhibition. Bioorg Med Chem. 2017;25(4):1364–1373. 28094224 10.1016/j.bmc.2016.12.043

[40] Pereyra CE, Dantas RF, Ferreira SB, Gomes LP, Silva-Jr FP. The diverse mechanisms and anticancer potential of naphthoquinones. Cancer Cell Int. 2019;19:207. 31388334 10.1186/s12935-019-0925-8 PMC6679553

[41] Shen X-B, Wang Y, Han X-Z, Sheng L-Q, Wu F-F, Liu X. Design, synthesis and anticancer activity of naphthoquinone derivatives. J Enzyme Inhib Med Chem. 2020;35(1):773–785. 32200656 10.1080/14756366.2020.1740693 PMC7144209

[42] Schepetkin IA, Karpenko AS, Khlebnikov AI, Shibinska MO, Levandovskiy IA, Kirpotina LN, Danilenko NV, Quinn MT. Synthesis, anticancer activity, and molecuodellingling of 1,4-naphthoquinones that inhibit MKK7 and Cdc25. Eur J Med Chem. 2019;183:111719–111732. 31563013 10.1016/j.ejmech.2019.111719 PMC6925601

[43] Mohamady S, Gibriel AA, Ahmed MS, Hendy MS, Naguib BH. Design and novel synthetic approach supported with molecular docking and biological evidence for naphthoquinone-hydrazinotriazolothiadiazine analogs as potential anticancer inhibiting topoisomerase-IIB. Bioorg Chem. 2020;96:103641. 32032844 10.1016/j.bioorg.2020.103641

[44] Biginelli P. Aldehyde- urea derivatives of aceto- and oxaloacetic acids. Gazz. Chim. Ital. 1893;23:360–413.

[45] Kambe S, Saito K, Kishi H, Sakurai A, Midorikawa H. A one-step of 4-oxo-2-thioxopyrimidine derivatives by the ternary condensation of ethyl cyanoacetate, aldehydes, and thiourea. Synth. 1979;1979(04):287–289.

[46] Mohamed MS, Awad SM, Ahmed NM. Synthesis and antimicrobial evaluation of some 6-aryl-5-cyano-2-thiouracil derivatives. Acta Pharm. 2011;61(2):171–185. 21684845 10.2478/v10007-011-0019-1

[47] Rami C, Patel L, Patel CN, Parmar JP. Synthesis, antifungal activity, and QSAR studies of 1,6-dihydropyrimidine derivatives. J Pharm Bioallied Sci. 2013;5(4):277–289. 24302836 10.4103/0975-7406.120078 PMC3831741

[48] Mourad A, Mourad M, Jones P. Novel HDAC/tubulin dual inhibitor: design,synthesis and docking studies of α-phthalimido-chalcone hybrids as potential anticancer agents with apoptosis-inducing activity. Drug Des Devel Ther. 2020;14:3111–3130. 10.2147/DDDT.S256756 PMC7425103 32848361

[49] Chen J. The cell-cycle arrest and apoptotic functions of p53 in tumor initiation and progression. Cold Spring Harb Perspect Med. 2016; 6(3):a026104. 26931810 10.1101/cshperspect.a026104 PMC4772082

[50] Shamas-Din A, Kale J, Leber B, Andrews D. Mechanisms of action of Bcl-2 family proteins. Cold Spring Harb Perspect Biol. 2013;5(4):a008714. 23545417 10.1101/cshperspect.a008714 PMC3683897

[51] Dewson G, Kluck RM. Mechanisms by which Bak and Bax permeabilise mitochondria during apoptosis. J Cell Sci. 2009;122(Pt 16):2801–2808. 19795525 10.1242/jcs.038166 PMC2736138

[52] Zhang Z, Lapolla SM, Annis MG, Truscott M, Roberts GJ, Miao Y, Shao Y, Tan C, Peng J, Johnson AE, et al. Bcl-2 Homodimerization Involves Two Distinct Binding Surfaces, a Topographic Arrangement That Provides an Effective Mechanism for Bcl-2 to Capture Activated Bax. J Biol Chem. 2004;279(42):43920–43928. 15302859 10.1074/jbc.M406412200 PMC1350923

[53] Lipinski CA, Lombardo F, Dominy BW, Feeney PJ. Experimental and computational approaches to estimate solubility and permeability in drug discovery and development settings. Adv Drug Deliv Rev. 2001;46(1-3):3–26. 11259830 10.1016/s0169-409x(00)00129-0

[54] Proudfoot JR. Drugs, leads, and drug-likeness: an analysis of some recently launched drugs. Bioorg Med Chem Lett. 2002;12(12):1647–1650. 12039582 10.1016/s0960-894x(02)00244-5

[55] Baviskar AT, Madaan C, Preet R, Mohapatra P, Jain V, Agarwal A, Guchhait SK, Kundu CN, Banerjee UC, Bharatam PV, et al. N-fused imidazoles as novel anticancer agents that inhibit catalytic activity of topoisomerase IIα and induce apoptosis in G1/S phase. J Med Chem. 2011; 54(14):5013–5030. 21644529 10.1021/jm200235u

[56] Liao JL. Molecular recognition of protein kinase binding pockets for design of potent and selective kinase inhibitors. J Med Chem. 2007;50(3):409–424. 17266192 10.1021/jm0608107

[57] Nandi S, Bagchi MC. In silico design of potent EGFR kinase inhibitors by structure based screening of combinatorial libraries. Mol Simul. 2011;37(3):196–209.

[58] Durgapal J, Bisht N, Dipiksha MA, Salman M, Nandi S. QSAR and structure-based docking studies of aryl pyrido[2,3-d]pyrimidin-7(8H)-ones. An Attempt to Anticancer Drug Design. IJQSPR. 2018;3(1):43–73.

[59] Nandi S, Bagchi MC. 3D-QSAR and molecular docking studies of 4-anilinoquinazoline derivatives: A rational approach to anticancer drug design. Mol Divers. 2010;14(1):27–38. 19330460 10.1007/s11030-009-9137-9

